# Maternally derived antibody titer dynamics and risk of hospitalized infant dengue disease

**DOI:** 10.1073/pnas.2308221120

**Published:** 2023-09-29

**Authors:** Megan O’Driscoll, Darunee Buddhari, Angkana T. Huang, Adam Waickman, Surachai Kaewhirun, Sopon Iamsirithaworn, Direk Khampaen, Aaron Farmer, Stefan Fernandez, Isabel Rodriguez-Barraquer, Anon Srikiatkhachorn, Stephen Thomas, Timothy Endy, Alan L. Rothman, Kathryn Anderson, Derek A. T. Cummings, Henrik Salje

**Affiliations:** ^a^Department of Genetics, University of Cambridge, Cambridge CB23EH, United Kingdom; ^b^Department of Virology, Armed Forces Research Institute of Medical Sciences, Bangkok 10400, Thailand; ^c^Department of Microbiology and Immunology, State University of New York Upstate Medical University, Syracuse, NY 13210; ^d^Department of Disease Control, Ministry of Public Health, Nonthaburi 11000, Thailand; ^e^University of California, San Francisco, CA 94143; ^f^Department of Cell and Molecular Biology, Institute for Immunology and Informatics, University of Rhode Island, Providence, RI 02903; ^g^Faculty of Medicine, King Mongkut’s Institute of Technology Ladkrabang, Bangkok 10520, Thailand; ^h^Department of Medicine, State University of New York Upstate Medical University, Syracuse, NY 13210; ^i^Coalition for Epidemic Preparedness Innovations, Washington, DC 20006; ^j^Department of Biology, University of Florida, Gainesville, FL 32611

**Keywords:** dengue, maternal antibodies, antibody dependent enhancement

## Abstract

Infants less than 1 y of age experience high rates of dengue disease in dengue virus (DENV) endemic countries. This burden is commonly attributed to antibody-dependent enhancement (ADE), whereby concentrations of maternally derived DENV antibodies become subneutralizing, and infection-enhancing. Understanding antibody-related mechanisms of enhanced infant dengue disease risk represents a significant challenge due to the dynamic nature of antibodies and their imperfect measurement processes. Further, key uncertainties exist regarding the impact of long-term shifts in birth rates, population-level infection risks, and maternal ages on the DENV immune landscape of newborns and their subsequent risks of severe dengue disease in infancy. Here, we analyze DENV antibody data from two infant cohorts (N = 142 infants with 605 blood draws) and 40 y of infant dengue hospitalization data from Thailand. We use mathematical models to reconstruct maternally derived antibody dynamics, accounting for discretized measurement processes and limits of assay detection. We then explore possible antibody-related mechanisms of enhanced infant dengue disease risk and their ability to reconstruct the observed age distribution of hospitalized infant dengue cases. We find that ADE mechanisms are best able to reconstruct the observed data. Finally, we describe how the shifting epidemiology of dengue in Thailand, combined with declining birth rates, have decreased the absolute risk of infant dengue disease by 88% over a 40-y period while having minimal impact on the mean age of infant hospitalized dengue disease.

## Significance

Our analysis represents a formal comparison of possible antibody-related mechanisms of severe infant dengue disease. We find that antibody-dependent enhancement mechanisms are best able to reconstruct age-specific patterns in infant dengue hospitalizations and quantify the titer-related relative risks of hospitalized infant disease. These estimates provide a path to monitoring age-specific dengue disease risk profiles in infants and can inform public health decisions and preventative measures. Further, we quantify the impact of changing dengue epidemiology and fertility trends in Thailand on maternal and infant dengue immune profiles and subsequent infant disease risks.

Dengue virus (DENV) is a mosquito-borne flavivirus with four serotypes (DENV1-4). As in many tropical regions, DENV is hyperendemic throughout South-East Asia. While primary DENV infections are typically mild or asymptomatic, secondary infections are associated with an increased risk of severe disease. Primary infections are believed to provide long-lasting protection against reinfection with that serotype and shorter-term protection against heterologous serotypes through the generation of cross-reactive antibodies (1). The increased disease risk associated with secondary infections is thought to be mediated by a mechanism known as antibody-dependent enhancement (ADE), whereby preexisting heterologous antibodies bind but do not neutralize infecting virions (2–4). Both antibody specificity and concentration are therefore thought to be key determinants of ADE and give rise to the idea of a titer-related “window” of risk. The risk window is believed to arise when cross-reactive antibody titer concentrations become low enough that they cannot effectively neutralize virions but high enough that they can enhance heterologous infections (3–5). Binding of nonneutralizing antibodies to the infecting serotype is believed to facilitate virus entry into Fc-receptor-bearing cells and subsequently, increase viral burden in the host (2, 6, 7).

ADE is also believed to occur in young infants when experiencing their primary infection if maternally derived anti-DENV antibodies are present (8–12). The placental transfer of IgG antibodies from mother to infant is an important mechanism for protecting young infants from infectious pathogens while the neonatal immune system is still developing (13). These transplacentally acquired antibodies decay within the first year of life, initially providing protection against infection, and subsequently waning over time to subneutralizing levels that have the potential to mediate ADE (illustrated in *SI Appendix*, Fig. S1). As antibody levels decay further, the ability to enhance the severity of heterologous infections is thought to be lost, though infants remain at risk of DENV infection. Peaks in reported dengue hospitalizations among infants less than 1 y of age are consistently observed in DENV-endemic countries and are widely attributed to maternal antibody-facilitated ADE (8, 12, 14). However, the specific concentrations of maternally derived DENV antibodies that may place infants at increased risk of severe disease have not been well characterized.

Quantifying the dynamics of maternally derived antibodies and associated risks of severe dengue disease requires carefully designed cohort studies with regular blood draws. However, very large cohort sizes are required to observe a sufficient number of severe dengue outcomes with which to directly investigate titer-associated risks. A further complication to our understanding of the relationship between preexisting DENV antibodies and risk of severe disease upon infection is that any antibody concentration-associated window of risk may be masked by lower limits of assay detection (typically a dilution of 1:10). For example, a previous study in Vietnam found that DENV-neutralizing antibodies declined to undetectable levels in >90% of infants by 6 mo of age (15). In addition, many assays measure antibody concentration as endpoint titers defined as the reciprocal of the highest sample dilution that gives a measurable effect. Typically conducted using twofold serial dilutions, this discretized measurement process obscures the true underlying continuous concentration values, adding to the challenge of disentangling antibody titer dynamics and associated risks.

Mathematical modeling can aid our understanding of these unobserved mechanisms. By accounting for assay characteristics such as the lower limit of detection and discretized measurements, the underlying temporal trends in antibody titers can be inferred. Given the immunological vulnerability of infants in their first year of life, there is a clear need to characterize the timing and duration of periods when they are at greatest risk of severe outcomes as well as to understand the mechanisms that mediate their risk. In this study, we use data from two longitudinal cohort studies conducted in Thailand which recruited infants at birth and measured DENV antibody levels at regular follow-up visits. We combine these data with the age distribution of infant dengue cases reported in local hospitals over a 40-y time period to explore the potential antibody-related mechanisms of infant risk of dengue hospitalization. Finally, we explore how changing trends in age-specific fertility rates and DENV transmission intensity in Thailand over the past 40 y may influence infant dengue disease risk profiles.

## Results

**Fig. 1.**
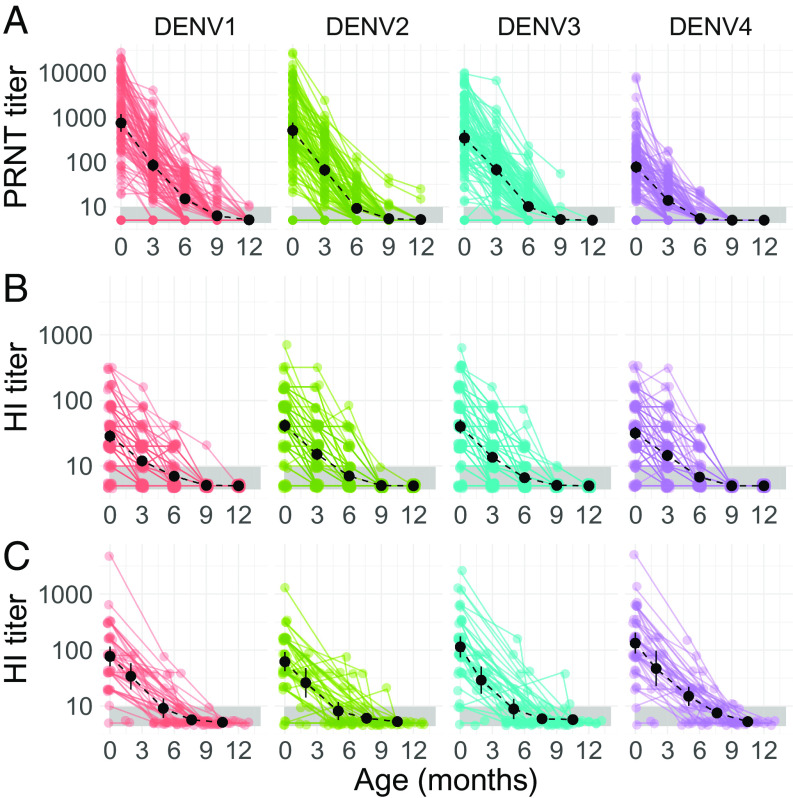
Infant DENV antibody titer trajectories by serotype. Measured DENV antibody titers by infant age observed in the Bangkok cohort (*A* and *B*) as measured by PRNT_50_ and HI assays, respectively, and in the Kamphaeng Phet cohort (*C*) measured by a HI assay. Titer values are shown on a log-linear scale in all panels. Colored points and lines show the observed titer trajectories of individual infants. Black points and lines represent the serotype-specific geometric mean titers and 95% CIs across infants in each age group. The black dashed line shows the trajectory of geometric mean titers across infants by age. The gray shaded area highlights the lower limit of assay detection (titers < 10) of the HI and PRNT assays.

We used longitudinal serological data from mother–infant cohorts in Bangkok (2000 to 2001) and Kamphaeng Phet (2015 to 2021), Thailand. We excluded all infant samples that had evidence of a DENV infection during the first year of life, as defined by an increase in mean DENV titers across serotypes at sequential blood draws. After exclusion of postinfection samples, any infant with ≥3 blood samples taken in the first year of life was included in our analysis (100 infants in Bangkok and 42 infants in Kamphaeng Phet, with a combined total of 605 blood draws). Geometric mean cord blood titers at the time of birth across the four serotypes in the Bangkok cohort were 316.7 (95% CI: 256.0 to 391.8) as measured by the plaque reduction neutralization test (PRNT_50_) and 34.9 (95% CI: 31.2 to 39.1) as measured by the hemagglutination inhibition (HI) assay (Fig. 1 *A* and *B*). Geometric mean PRNT_50_ titers fell to 47.8 (95% CI: 40.8 to 56.0) by 3 mo and to 9.3 (95% CI: 8.5 to 10.3) by 6 mo, by which time 63.1% of PRNT_50_ titers were below the limit of detection. Mean HI titers fell to 13.8 (95% CI: 12.5 to 15.1) at 3 mo and to 6.9 (95% CI: 6.4 to 7.3) at 6 mo. By 12 mo, 98.9% of PRNT_50_ and 100% of HI measurements were below the limit of detection. A similar pattern was observed in the Kamphaeng Phet cohort, with geometric mean cord blood HI titers of 92.4 (95% CI: 75.2 to 113.5), falling to a mean of 33.2 (95% CI: 25.1 to 44.0) by 3 mo and 10.0 (95% CI: 8.3 to 12.1) by 6 mo (Fig. 1*C*). At 6 mo, 57.3% of titers were below the limit of detection, rising to 93% at 12 mo. Antibody titers in mothers’ sera were strongly correlated with cord blood titers (Pearson correlation coefficient, R = 0.94 in Bangkok for PRNT_50_, and 0.87 for HI in Bangkok) (*SI Appendix*, Fig. S2). Cord blood titers were also strongly correlated with infant serum titers at ages <6 mo, though the correlation decreased with increasing infant age (*SI Appendix*, Fig. S2).

**Fig. 2.**
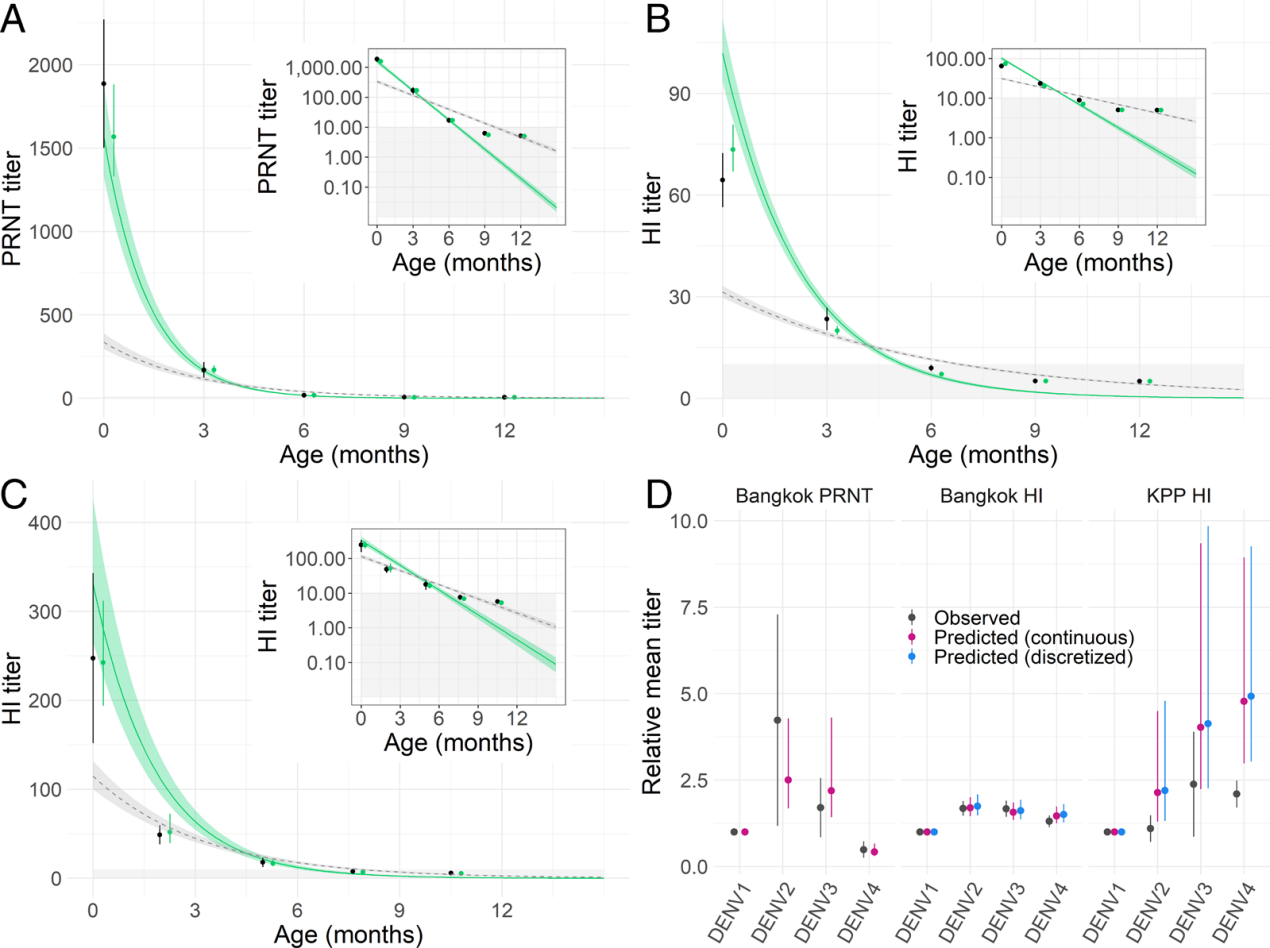
Observed and predicted population DENV antibody titers in infants. Panels *A*–*C* show model-reconstructed infant population DENV titers for the Bangkok (*A* and *B*) and Kamphaeng Phet (*C*) cohorts. The black points and lines show the mean and 95% CI of observed infant titers by age. Green points and vertical lines show model median and 95% CrI estimates of mean infant titers by age, where estimated titers were transformed to match the discretized measurement process and lower limit of detection truncation. The green lines and shaded ribbons represent the median and 95% CrI estimates of the underlying continuous-scale population mean DENV titers. Gray dashed lines and shaded ribbons show the median and 95% CrI estimates of population mean DENV titers using the “naive” cohort model, where the discretized measurement process and lower limits of assay detection are not accounted for. The *Insets* in panels *A*–*C* show the same model fits using a log-linear scale for antibody titers. Grey shaded regions represent titer values below the assay limits of detection. The mean titer of each serotype, relative to DENV1, by cohort and assay are shown in panel *D* where the black points and lines show the observed mean and 95% CI, and colored points and lines represent the model median and 95% CrI estimates.

We developed a mathematical model to reconstruct maternally derived antibody titers over the first 12 mo of life in the two cohorts and explored a range of model parameterizations. The best-fitting model, as assessed by the widely applicable information criterion (WAIC), assumed time-constant antibody decay rates and estimated independent decay rates for each assay and cohort (*SI Appendix*, Table S1). We estimated a median half-life of 28.1 d [95% credible interval (CrI): 27.1 to 29.1] for PRNT_50_ titers in the Bangkok cohort, a half-life of 47.0 days (95% CrI: 44.7 to 49.5) for HI titers in the same cohort and a half-life of 38.5 days (95% CrI: 35.6 to 41.4) for HI titers in the Kamphaeng Phet cohort. For the remainder of the results, we focus on estimates from this best-performing model. The estimated population-level maternally derived antibody dynamics are shown by the solid green lines in Fig. 2 *A–C*. We compare these estimates to the observed data at each time point, accounting for the discretized measurement process of the assay (HI) and the lower limits of assay detection, shown by the black (observed) and green (estimated) points in Fig. 2 *A*–*C*. Observed vs. predicted titers and model fit to serotype-specific data are shown in *SI Appendix*, Figs. S3 and S4. We observe significant heterogeneity in antibody titer concentrations across serotypes, with no clear trends by cohort or assay (Fig. 2*D*). We conducted sensitivity analysis on the exclusion criteria of possible infant infections, where infant samples with increases in serotype-specific titers were additionally excluded from the analysis. We find that 79 infants had increases in serotype-specific titers, compared to 40 when looking at increases in mean titers across serotypes. We found no significant differences in antibody half-life estimates using these different exclusion criteria, shown in *SI Appendix*, Fig. S5 and Table S2.

A total of 1,434 infant dengue cases ≤12 mo of age were reported at Queen Sirikit National Institute of Child Health (QSNICH) in Bangkok between 1974 and 2014 and 84 cases at Kamphaeng Phet (KPP) hospital between 1994 and 2019 (*SI Appendix*, Figs. S6 and S7). The mean age of reported infant (≤12 mo) dengue hospitalizations was 7.3 (95% CI: 7.1 to 7.4) mo in QSNICH and 7.7 (7.0 to 8.3) mo in KPP hospital. Using the risk of infant hospitalization in 12-mo-olds as a reference point and assuming a constant risk of exposure for all infants ≤12 mo, we estimate the relative risk (RR) of infant hospitalization with dengue to range from 0.2 (95% CI: 0.1 to 0.1) in 1-mo-old infants to 4.4 (95% CI: 3.2 to 6.1) in 8-mo-old infants (Table 1). There was no significant difference in the mean age of infant dengue cases by sex or by month of hospitalization (*SI Appendix*, Fig. S8), though the mean age of infant DENV-4 cases was lower than that of other serotypes (*SI Appendix*, Fig. S8).

**Table 1.** RR of infant dengue hospitalization by month of age, relative to the risk in 12-mo-old infants

Age (months)	Relative risk (95% CI)
1	0.19 (0.09 to 0.40)
2	0.47 (0.27 to 0.79)
3	0.98 (0.64 to 1.48)
4	1.95 (1.36 to 2.82)
5	2.93 (2.01 to 4.13)
6	4.00 (2.87 to 5.57)
7	3.67 (2.63 to 5.13)
8	4.42 (3.18 to 6.14)
9	3.44 (2.46 to 4.82)
10	2.33 (1.63 to 3.32)
11	1.81 (1.25 to 2.63)
12	1.00

**Fig. 3.**
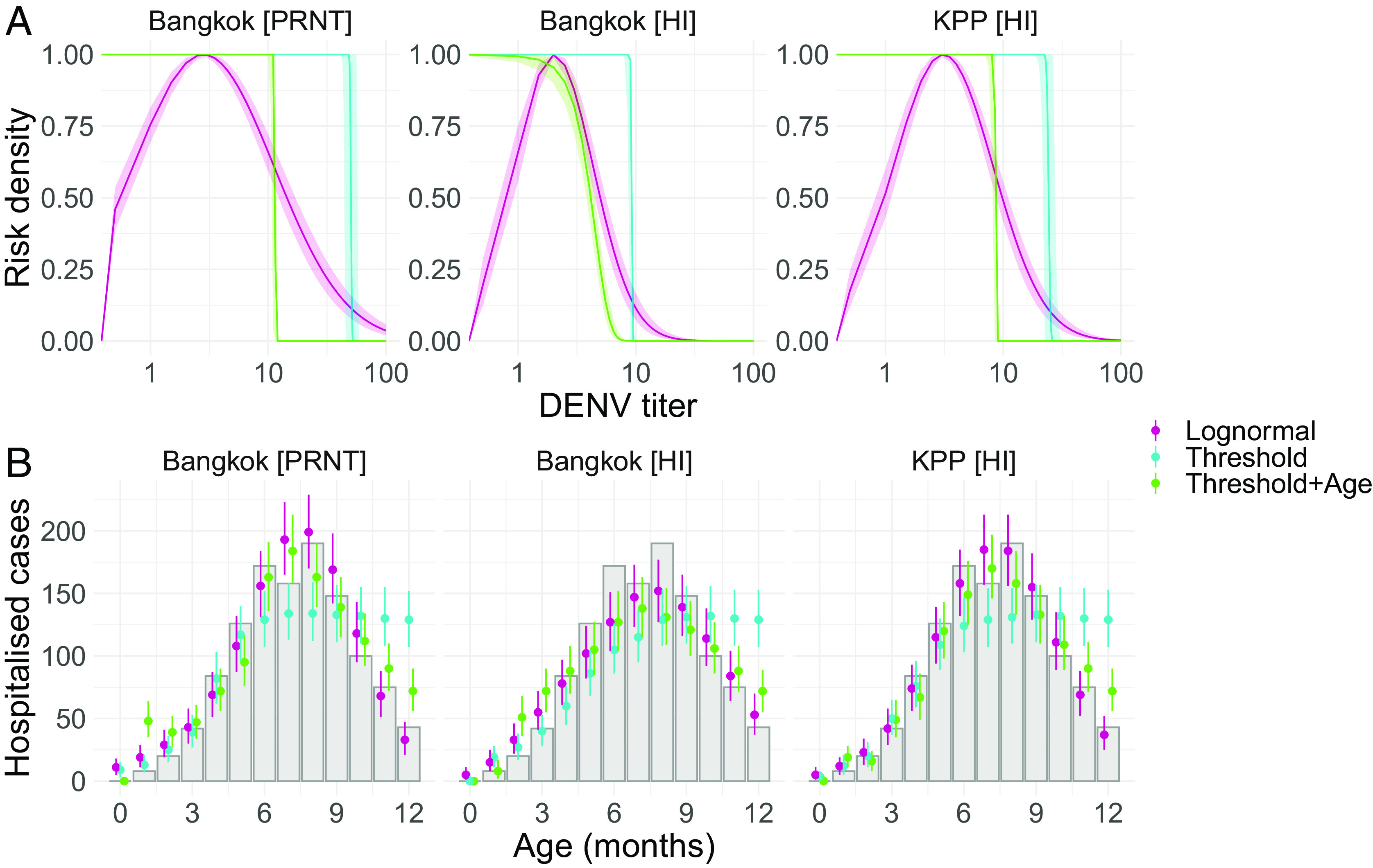
Antibody-related mechanisms of infant disease and associated age-specific risk profiles. Panel *A* shows model estimates of DENV titer-related relative risks and risk thresholds for hospitalized infant dengue disease in each of the cohorts and assays. The pink lines and shaded ribbons show the median and 95% CrI titer-risk estimates from the model assuming a log-normal relationship between DENV titers and enhanced risk of hospitalized infant dengue disease upon infection. The blue vertical lines and shaded ribbons show the median and 95% CrI protective titer threshold estimates from the threshold model, while the green vertical lines and ribbons show median and 95% CrI estimates of the protective titer threshold from the threshold model with estimated age-specific disease frailty (δ). Panel *B* shows the observed and predicted age distribution of hospitalized infant dengue cases, between 0 and 12 mo of age, for each of the considered models. The gray bars show the cumulative number of reported infant dengue hospitalizations by age across the two hospitals. Colored points and lines show the median and 95% CrI estimates of age-specific hospitalized infant dengue cases reconstructed by each model.

To explore the potential mechanistic relationships between maternal DENV titers and enhanced infant dengue disease risk, we use the previously estimated maternally derived DENV titers on a continuous scale to reconstruct expected numbers of age-specific hospitalized infant dengue cases using a range of model parameterizations. We consider two main scenarios for the relationship between maternally derived DENV antibodies and risk of hospitalized dengue disease. We first consider a mechanism consistent with ADE, whereby infant risk of hospitalized dengue disease, given a DENV infection, varies as a function of DENV antibody concentration, following either a normal or lognormal functional form. In the second scenario, we consider a threshold mechanism where DENV antibody concentrations determine only susceptibility to infection. Here, infants with DENV antibody concentrations above a threshold are protected from infection. Below the threshold, all infants are equally susceptible to infection and have a constant risk of hospitalized dengue disease upon infection, independent of antibody concentrations (non-ADE scenario). For both of these scenarios, we also consider model versions where infant vulnerability to hospitalized disease given a DENV infection declines exponentially from birth as a function of infant age, consistent with underlying frailty decreasing over the first year of life (16, 17). We find that the ADE scenario is able to reconstruct the observed age distribution of hospitalized infant dengue cases, both with and without an age-specific disease vulnerability effect (*SI Appendix*, Table S3 and Fig. 3 *A–C*). The performance of the ADE models, as assessed by the WAIC, increased with decreasing values of δ (the monthly rate of decline in age-specific vulnerability to hospitalized disease), resulting in increasing estimates of mean risk titers (*SI Appendix*, Table S3). The ADE models with no age-specific disease vulnerability effect performed better than those with the effect (*SI Appendix*, Table S3). While both the normal and lognormal ADE functional forms were able to reconstruct age-specific trends in hospitalized dengue cases (Fig. 3 *A*–*C*), the lognormal distribution was consistently preferred by the WAIC (*SI Appendix*, Table S3 and Fig. S9). This is consistent with the observed relationship between the number of age-specific hospitalized infant cases and the expected mean titers at the corresponding ages, which are approximately lognormal distributed (*SI Appendix*, Fig. S10). For the non-ADE threshold scenario, we find that this mechanism could broadly reconstruct observed age-specific hospitalized disease trends only with the addition of the age-specific disease vulnerability effect (Fig. 3 and *SI Appendix*, Fig. S9). The best-performing non-ADE model estimated a δ of 0.21 (0.19 to 0.22) per month (*SI Appendix*, Table S3). However, the non-ADE threshold models were consistently outperformed by the ADE models as compared by the WAIC (Fig. 3 and *SI Appendix*, Table S3).

**Fig. 4.**
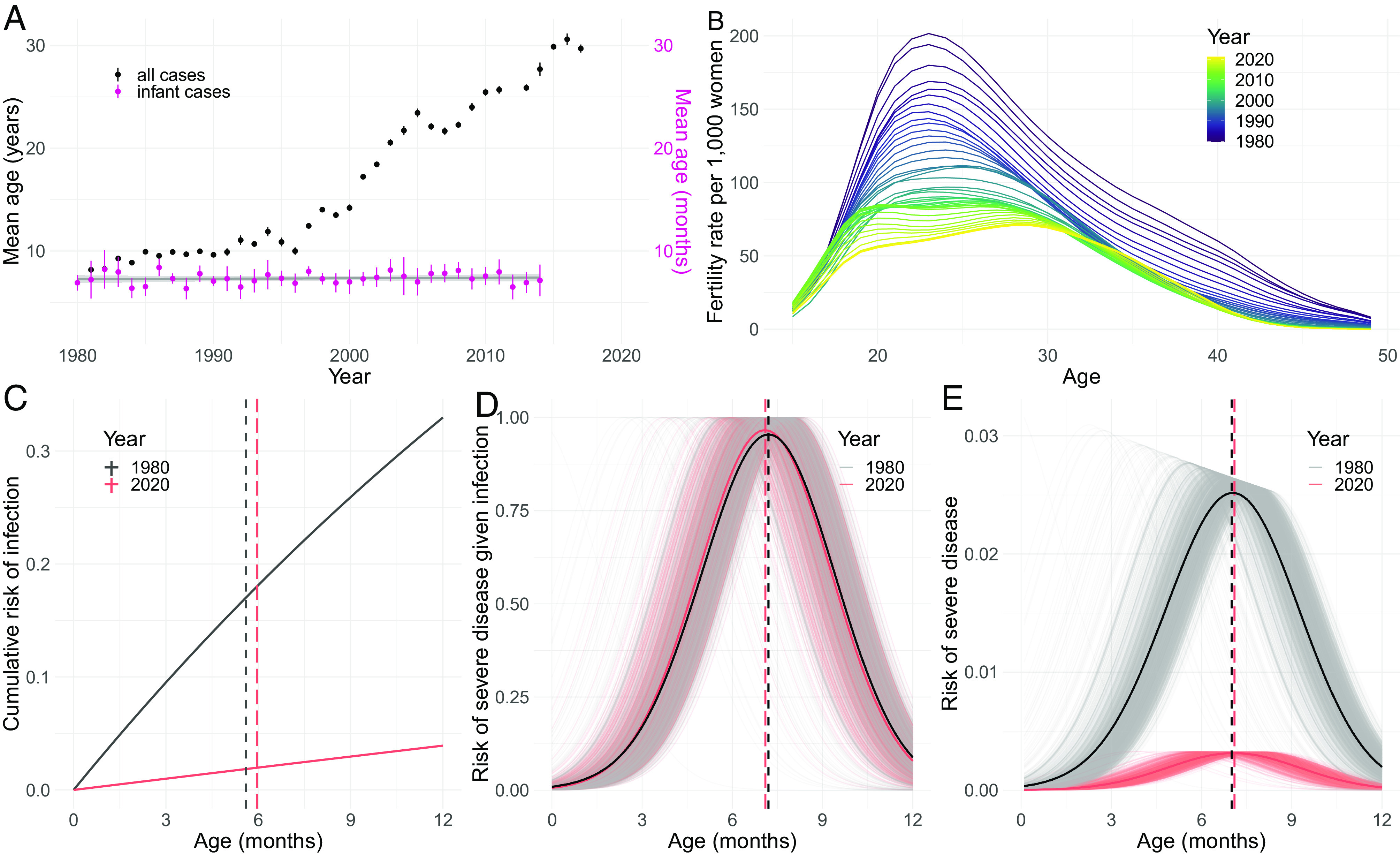
Impact of the shifting epidemiology of DENV on infant risk profiles. (*A*) Black points and lines show the mean age of reported hospitalized dengue cases in Bangkok from 1981 to 2017 with 95% CIs. The violet points and lines show the mean age in months of hospitalized dengue cases among infants ≤1 y reported in QSNICH in Bangkok and Kamphaeng Phet Hospital between 1980 and 2019. (*B*) Annual age-specific fertility rates in Thailand from 1980 to 2020 (18). (*C*) The cumulative age–specific risk of infant DENV infection in the years 1980 and 2020 are shown by the black and pink solid lines, respectively. The vertical dashed black and pink lines show the expected mean age of infant infections for 1980 and 2020, respectively. (*D*) The estimated population risks of severe infant dengue disease given infection in the years 1980 and 2020 are shown by the black and pink solid lines, respectively. Shaded gray and pink lines show the individual-level risks of severe infant dengue disease given infection from the simulation for 1980 and 2020, respectively. The vertical dashed black and pink lines show the expected mean age of severe infant disease risk given a DENV infection for 1980 and 2020, respectively. (*E*) The estimated population risk of severe infant dengue disease in the years 1980 and 2020 are shown by the black and pink solid lines, respectively. Shaded gray and pink lines show the individual-level risks of severe infant dengue disease from the simulation for 1980 and 2020, respectively. Vertical dashed black and pink lines show the expected mean age of severe infant disease risk for 1980 and 2020, respectively.

Finally, we explore the potential effects of long-term changes in the epidemiology of DENV and shifting demography in Thailand on age-specific infant dengue risk profiles. We focus on the ADE scenario that assumes a lognormal functional form with no age-specific disease frailty effect. A decline in the force of DENV infection over the last 40 y has led to an increasing mean age of reported dengue cases in Bangkok from 8.2 y in 1981 to 29.7 y in 2017 (Fig. 4*A*). However, the mean age of infant dengue hospitalizations (aged ≤1 y) has remained stable over this same time frame (*P*-value from linear regression for trend = 0.95). During this 40-y period, fertility rates in Thailand have declined from an average of 3.4 children per woman in 1980 to 1.3 per woman in 2020, while the mean age of childbirth has remained relatively stable from 28.7 in 1980 to 28.1 y in 2020 (18). Using annual age-specific fertility rate data (Fig. 4*B*) and assuming a linear decline in DENV transmission intensity from 0.4 in 1980 to 0.04 in 2020 (18–20), we simulate the expected trends in infant dengue risk profiles over this 40-y period. We estimate a 13.2% reduction in the proportion of mothers who have experienced ≥2 DENV infections at the time of birth—from an estimated 99.7% of mothers in 1980 to 86.5% of mothers in 2020 (*SI Appendix*, Fig. S11).

We show that under these assumptions, the cumulative probability of experiencing a DENV infection by 1 y of age declined from 0.33 in 1980 to 0.04 in 2020. However, the mean age of infant infection increased only slightly, from 5.6 to 5.9 mo over the same period (Fig. 4*C*). We further estimate that the reduced immune levels in birthing women, driven by decreasing force of infection (FOI) and minor demographic shifts, had a limited effect on the mean age of infant titer-related risk of hospitalized dengue disease, from 7.2 mo in 1980 to 7.1 mo in 2020 (Fig. 4*D*). Subsequently, this combination of reduced mean age of hospitalized disease risk and increased mean age of infection led to a stable mean age of infant hospitalized dengue disease occurrence during this 40-y period (mean age of 7.1 mo between 1980 and 2020) (Fig. 4*E*). Despite the stable mean age of hospitalized disease among infants, we estimate an 87.7% reduction in absolute risk of hospitalized infant dengue disease between 1980 and 2020 under these assumptions (Fig. 4*E*). This is consistent with reported dengue case data over this same time period where the proportion of all dengue cases in Bangkok attributed to infants <1 y of age has declined significantly from 3.2% in 1981 to 0.6% in 2017 (*SI Appendix*, Fig. S12).

## Discussion

We use mathematical models to investigate the relationship between infant maternally derived DENV antibody titers and the associated risks of hospitalized dengue disease, using detailed longitudinal serological data and dengue hospitalization data. By probabilistically reconstructing maternally derived DENV antibody titer distributions at the ages of enhanced risk of infant dengue hospitalization, our analysis provides an improved understanding of the possible mechanisms that underpin this relationship and their respective quantitative correlates of risk.

Our analysis highlights the valuable role that mathematical models can play in scenarios of poorly observed processes. Our results show the substantial inferential biases that can occur when discretized measurement processes and lower limits of assay detection are not accounted for. Even in the case of the PRNT_50_ assay that measured antibody concentrations on a continuous scale, not accounting for the lower limit of assay detection where the observed data become truncated results in large biases in antibody half-life estimates as well as poor fits to the observed data (Fig. 2*A* and *SI Appendix*, Table S1). Accounting for these measurement processes, we infer the underlying dynamics of maternally derived mean DENV antibody titers in Thai infants and find that constant exponential decay models are best able to capture the observed infant antibody dynamics in this setting (Fig. 2 *A–C* and *SI Appendix*, Table S1 and Fig. S13). Our estimates of infant DENV antibody half-lives, ranging from a median of 28 to 47 d across cohorts and assays, are consistent with previous estimates of 41 d estimated in Thai infants (21), serotype-specific antibody half-lives ranging from 33 to 53 d in Thai infants (22), and in Vietnamese infants with an estimated half-life of 42 d (14).

We find that mechanisms of infant dengue disease enhancement directly driven by maternally derived DENV antibody concentrations (ADE scenario) give rise to age-specific patterns in infant hospitalizations that are consistent with observed data. We show that the threshold (non-ADE) scenario alone could not reconstruct observed age patterns of infant dengue hospitalizations. However, with the addition of an age-specific infant disease vulnerability effect in the non-ADE model, the reconstructed age distribution becomes more similar to observed trends, though is consistently outperformed by ADE models as compared by the WAIC (*SI Appendix*, Table S3). Further, the non-ADE models estimate protective titer thresholds below 40, though DENV infections have been shown to frequently occur at much higher titer concentrations (23). It is important to note that our analysis focuses on DENV antibody concentration using mean titers across serotypes and is not able to investigate the potential role of antibody specificity in infant disease risk. The roles of DENV antibody concentration and serotype specificity are highly intertwined with antibodies against one serotype able to effectively neutralize other serotypes at high enough concentrations. Disentangling the roles of DENV antibody concentration and specificity in disease risk therefore represents a significant challenge in endemic settings such as Thailand, where all four serotypes cocirculate and where a majority of exposures are not observed. Further research will be needed to understand the representativeness of our findings to other settings, particularly in nonendemic regions, and to investigate the varying roles of antibody concentration and specificity in infant dengue disease risk.

Our estimated distribution of HI titer concentrations in the ADE scenario that are associated with the ages of enhanced risk of infant hospitalization in the Kamphaeng Phet cohort is similar to the HI titers estimated to enhance the risk of hospitalization in older children and adults experiencing postprimary infections, with slightly lower risk-associated concentrations estimated for infants (*SI Appendix*, Fig. S14) (5). Further studies are needed to understand whether the differences between the two estimated distributions in this setting are true differences in immune responses or are perhaps explained by assay noise or differences in the antigenic properties of the circulating viruses in the two cohorts, which occurred at different time periods (2015 to 2021 and 1998 to 2003) (5, 24, 25). We note that unlike older children and adults experiencing postprimary infections, infants lack preexisting B cell or T cell immunity, suggesting a potentially different role of antibodies in immune response to infant DENV infections. While PRNTs measure neutralization, HI assays measure antibodies that bind to virions and inhibit hemagglutination but are not necessarily neutralizing. However, we observe a good correlation between PRNT_50_ and HI titer values with a Pearson correlation coefficient, r = 0.62 (*SI Appendix*, Fig. S15), and find largely consistent titer-associated risk windows inferred independently from the PRNT_50_ and HI data. We also observed a trend of an increasing HI:PRNT ratio with increasing infant age in the Bangkok cohort, suggesting a potentially increased role of binding and HI as neutralization capabilities decrease, though further work is needed to understand the drivers of this trend (*SI Appendix*, Fig. S16).

Our study highlights the complications caused by the nonstandardization of antibody assays, where differences in the estimated titer windows of risk across cohorts/locations may be due to differences in the antigenic properties of the specific viruses included in each assay rather than due to fundamental differences in risk by serotype-specific titers (26, 27). This presents a significant challenge when comparing inferences across locations and studies where both differences in the circulating lineages and differences in reference viruses used in the assay can impede direct comparisons of results. We note that infant cases caused by DENV-4 had a lower mean age compared to cases caused by other serotypes and that DENV-4 also had the lowest neutralization titers (but highest HI titers). However, given the reliance of titer measurements on the virus used in the assay, we cannot draw any mechanistic conclusions with these data alone. We also note that despite the seasonality of DENV transmission in Thailand, we observe no significant differences in the mean age of infant dengue hospitalizations by month (*P*-value for linear trend = 0.63), suggesting minimal effects of seasonal transmission intensity on infant disease risk (*SI Appendix*, Fig. S8).

A steady decline in DENV force of infection in Thailand over the last 40 y has been well documented (19, 20, 28), leading to an increasing mean age of dengue cases. This trend has been driven, at least in part, by simultaneously decreasing fertility rates, which has led to reduced numbers of susceptible individuals in the population (19). We demonstrate how reduced transmission intensity of DENV over time has also shifted the immune landscape such that women of childbearing age have experienced fewer DENV infections than in previous decades, leading to reduced levels of transplacentally transferred DENV antibodies in newborns. We show how these demographic changes have had a negligible impact on the mean age of infant hospitalized cases, despite a substantial reduction in DENV transmission. However, we find that reduced transmission intensity, coupled with declining fertility rates, has driven a large reduction in the absolute risk of hospitalized dengue disease posed to infants. It remains critical, however, to shield infants at ages where they are at enhanced risk of severe dengue disease, for example, through limiting exposure to mosquitoes by placing screens on doors/windows and/or the use of long-sleeved clothing (29, 30). Further, careful consideration should be given to the risk profile of young infants when planning the implementation of any future DENV vaccines. The potential long-term effects of DENV vaccines on infant risk of severe disease, through both changes in mothers’ antibody levels and changes in transmission intensity, should be assessed, particularly in the case where vaccines induce lower titers than natural infection (31).

The cohort studies used in this analysis were not designed to investigate individual-level infant risk of severe dengue disease. Doing so would require very large cohort sizes in order to observe a substantial number of severe outcomes. Our estimates of titer concentrations associated with increased risk of hospitalized dengue are derived from population-level probabilistic inferences rather than individual-level observations, assuming that the infants recruited to the cohort studies are representative of the wider infant population. In addition, uncertainty from model estimates of maternally derived DENV antibody concentrations was not propagated through to the models estimating titer concentrations associated with the age of enhanced hospitalization risk. The CrI estimates from this model therefore do not reflect the full underlying uncertainty. We also assume the population age distribution of infants aged <1 y to be uniform by month. Despite these simplifying assumptions, our findings are consistent with a study of 13 Thai infants with dengue hemorrhagic fever or dengue shock syndrome that were estimated to have PRNT_50_ titers <10 at the time of infection (12). In the simulation analysis, we assume the mother–infant cohorts to be representative of the wider Thai population. However, the mean age of birthing women in the two cohorts are slightly younger (25.0 in Bangkok and 25.2 in Kamphaeng Phet) than that of the wider Thai population at 27.5 y (*SI Appendix*, Fig. S17). Our study highlights the need for additional focused mother–infant cohort studies with more frequent antibody measurements as well as active surveillance of DENV infections and associated illness occurring in the first year of life.

Our analysis has allowed an increased understanding of the dynamics of infant dengue disease risk, including the potential epidemiological and demographic drivers that shape these risk profiles. By probabilistically reconstructing the distribution of dengue antibody titer concentrations that place infants at enhanced risk of hospitalized disease given infection, we have provided an estimate of antibody-based correlates of risk that can inform control and prevention efforts. As age-specific risks of infant dengue disease depend on local DENV transmission and immune dynamics, the characterization and monitoring of local maternal DENV immune profiles in endemic countries will be crucial for informing maximal preventative and control measures.

## Methods

### Cohort Data.

We use DENV serological data from two longitudinal cohort studies in Thailand. The first study was an infant cohort study conducted in Bangkok from 2000 to 2003 (32). A total of 219 mother–infant pairs attending the Rajavithi Hospital in Bangkok between November 2000 and March 2001 were enrolled in the cohort (32, 33). Mother and cord blood samples were collected at the time of birth. Of these 219 infants, 138 (63%), 116 (53%), 118 (54%), and 115 (53%) infants were followed up at 3, 6, 9, and 12 mo of age, respectively, where blood was drawn for serological testing. All blood samples were tested using DENV serotype–specific HI and 50% PRNT_50_ assays (32). PRNT_50_ titers were measured on a continuous scale with a lower limit of detection of 10, while HI titers were measured on a discretized scale using twofold serial dilutions with a lower limit of 1:10. Mothers with a history of immune deficiency, who received immunosuppressive treatment in the previous month, or those who received blood in the previous 3 mo were excluded from study participation. Premature infants, twins, or infants with severe congenital anomalies were also excluded from the study (32).

The second cohort study is an ongoing family cohort in Kamphaeng Phet, Thailand (25). Mother–infant pairs enrolled between September 2015 and July 2021 were included in our analysis. Blood was drawn from each mother prior to giving birth as well as cord blood at the time of birth. Infants were followed up at approximately 1 y of age where blood samples were taken. Additional blood samples were collected from a subset of infants in the first year of life as part of household-illness investigations within the cohort. DENV antibody titers were tested using serotype-specific HI measured on a discretized scale using twofold serial dilutions with a lower limit of detection of 1:10.

To account for possible infection-related increases in antibody titers, the time at which any mean titer increase (mean across serotypes) was observed in an infant was marked as a potential DENV infection. Titer measurements from these and any subsequent samples of the same infant were then excluded from the main analysis. Sensitivity analyses were conducted where infant samples with serotype-specific titer rises were additionally excluded from the analysis. Samples with measured concentrations below the lower limit of assay detection (1:10) were assigned a value of 5 for the calculation of geometric mean titers by age. Infants who provided at least three samples within the first 13 mo and who had no evidence of a DENV infection were included in the final analysis. A total of 142 infants were included in the final analysis, with 42 infants from the Kamphaeng Phet cohort and 100 infants from the Bangkok cohort representing a combined total of 605 blood samples.

### Dengue Hospitalization Data.

To explore the age-specific risks of hospitalized infant dengue disease, we used reported hospitalized dengue case data from QSNICH, located in Ratchathewi, Bangkok, reported between 1974 and 2014, as well as from Kamphaeng Phet hospital, located in Kamphaeng Phet province, between 1994 and 2019. Hospitalized cases with any dengue diagnosis (dengue fever or dengue hemorrhagic fever) confirmed by PCR or serological testing and with age information were included in the analysis. Both hospitals are large, tertiary care facilities that accept patients from throughout their respective provinces.

### Estimating Infant Maternally Derived Antibody Titers.

We model the decay of maternally derived DENV antibody titers from birth until 13 mo of age. To account for the discretized measurement process of the HI assay as well as the lower limit of detection truncation of both the HI and PRNT assays, we consider “true” unobserved continuous-scale antibody titers as distinct but related quantities to the measured antibody titers. Twofold dilutions of 1:10, 20, 40, 80, 160, and so on were used for HI assay measurements. As HI antibody concentrations are reported as the lower bound of the dilution (e.g., antibody concentrations in the range of 1:80 to 1:159 will be reported as 1:80), we consider the “true” antibody concentration, $A_{i,s,a}$, for an individual, i, to serotype, s, at age, a, on a continuous scale that underlies the discretized observed concentration, $A_{i,s,a}^{*}$, measured by the assay (5, 31). Analysis of titers was conducted on an adjusted $\log_{2}$ scale; $1+\log\left(A_{i,s,a}^{*}/10\right)/\log\left(2\right)$, such that a measured titer of 1:10 = 1, 1:20 = 2, 1:40 = 3, and so on. We define the probability of the observed titer concentration, given the true concentration as shown in Eq. **1**, where f(x) is a cumulative probability density function of a normal distribution, with estimated SD σ.

[1] $$P\left(A_{i,s,a}^{*}|A_{i,s,a}\right)=\int_{A_{i,s,a}^{*}}^{A_{i,s,a}^{*}+1}f\left(x\right)dx,$$

[2] $$A_{i,s,a}=A_{i,s,a=0}\;\exp\left(-\gamma a\right),$$

[3] $$\text{if}\;(a\leq \tau )\;A_{i,s,a}=A_{i,s,a=0}\;\text{exp}\left(-\gamma_{1}\tau \right),$$

[4] $$\begin{array}{c} {\mathrm{if}\left(a>\tau \right)\;A}_{i,s,a}=A_{i,s,a=0}\;\exp\left(-\gamma_{1}\tau \right)\;\exp\left(-\gamma_{2}\left(a-\tau \right)\right), \end{array}$$

[5] $$L=\sum^{i,s,a,z}P\left(A_{i,s,a,z}^{*}|A_{i,s,a,z}\right).$$

The initial “true” (unobserved) antibody titer of each infant was estimated, and the “true” titers of each subsequent sample (including those below the lower limit of detection) were inferred on a continuous scale assuming either constant or biphasic rates of decay. Where a constant rate of decay was assumed, “true” antibody titers were calculated as shown in Eq. **2**, where γ is the estimated rate of decay and a is infant age at the time of sample collection. For biphasic decay dynamics, the age at which the rate of decay changes, τ, was additionally estimated, and “true” titers were estimated as shown in Eqs. **3** and **4**. The full model likelihood was evaluated by summing the probability of observed titers given the estimated “true” titers across individuals, i, serotypes, s, ages, a, and cohorts and assays, z, as shown in Eq. **5**.

We consider a number of model parameterizations. We consider a “base” model, where rates of antibody decay are assumed to be constant across different assays and cohorts, and a “cohort” model where rates of antibody decay are allowed to vary by assay and cohort. In addition, we consider model versions with constant decay rates across the four serotypes and with serotype-specific decay rates. Finally, we also consider a “naive” model, where the discretized measurement processes and lower limits of assay detection are not accounted for in the model. The WAIC was calculated for each model specification to compare model performance. We fit exclusively to infant samples taken between birth (cord blood) and 13 mo of age. All model fitting was conducted in CmdStanR version 0.3.0. Each model was run with four chains of 5,000 iterations each, with a warm-up period of 1,000 iterations.

### Modeling Titer-Related Mechanisms of Enhanced Infant Dengue Disease Risk.

We combine the median estimates of “true” continuous-scale infant antibody titers with the observed age distribution of reported hospitalized infant dengue cases to explore potential titer-related mechanisms of disease risk. Here, we assume that dengue antibody titer measurements within the two cohorts are representative of their respective populations. We consider two main mechanisms that relate antibody titers to disease risk. First, we consider a mechanism consistent with ADE, whereby infant risk of hospitalized dengue disease given a DENV infection peaks at some intermediate antibody concentrations and declines at concentrations above and below this value. This relationship was modeled assuming either normal or lognormal function forms. For the second, alternative non-ADE scenario, we consider a threshold mechanism where DENV antibody concentrations determine only susceptibility to infection. Here, infants with antibody concentrations below a threshold will be equally susceptible to infection and have a constant risk of hospitalized dengue disease given a DENV infection independent of antibody concentration. For both of these scenarios, we also consider model versions with an added effect of age-specific vulnerability to severe disease (16, 17), where infant vulnerability to hospitalized disease declines exponentially from birth with rate δ.

For each considered scenario, we calculate the expected number of hospitalized infant dengue cases by month of age, ${\overset{}{C}}_{a}^{}$, as shown in Eqs. **6** and **7**. Eq. **7** represents the model version assuming age-specific frailty to severe dengue disease given a DENV infection. Here, λ is the monthly DENV transmission intensity which we assume to be endemic and constant in time with a rate of 0.01 per month (0.12 per year). f() represents a probability density function of a normal or lognormal distribution in the ADE scenario and a cumulative normal distribution for the non-ADE threshold scenario, with estimated mean, μ, and SD σ. Probability density values were rescaled to RR density values by dividing by the distribution mode. β is the cumulative infant population size, and N is the number of infants included in the cohort study. Models were fit with fixed values of δ at 0.05, 0.15, and 0.25 and with an estimated δ.

[6] $${\hat{C}}_{a}=\frac{\beta }{N}\sum^{i}\int_{a-1}^{a}\left(1-e^{-\lambda a}\right)\;f\left(A_{i,a}|\mu ,\;\sigma \right),$$

[7] $$\begin{array}{c} {\hat{C}}_{a}=\frac{\beta }{N}\sum^{i}\int_{a-1}^{a}\left(1-e^{-\lambda a}\right)\;f\left(A_{i,a}|\mu ,\;\sigma \right)e^{-\delta a}, \end{array}$$

[8] $$L=\prod_{a=1}^{N}\frac{{{\hat{C}}_{a}}^{C_{a}}\exp\left(-{\hat{C}}_{a}\right)}{C_{a}!}.$$

Age-specific counts of hospitalized infant dengue cases were assumed to follow a Poisson distribution with the model likelihood calculated as shown in Eq. **8**. Model fitting was conducted in CmdStanR version 0.3.0. Each model was run with four chains of 5,000 iterations each including a warm-up period of 1,000 iterations. The WAIC was used to compare model performance.

### Modeling Temporal Trends in Infant Risk of Hospitalized Dengue Disease.

We use data on annual age-specific fertility rates in Thailand for the years 1980 to 2020 from the United Nations World Population Prospects (18). We assume that the transmission intensity of DENV in Thailand declines linearly from 0.4 to 0.04 between 1980 and 2020, as estimated in previous analyses (19, 20), and that it was constant at 0.4 in years prior to 1980. Given the age-specific number of birthing women each year, we calculate their probabilities of being susceptible, monotypic, and multitypic to DENV as shown in Eqs. **9** and **10**.

[9] $$P\left(\mathrm{Susceptible}\right)=\exp\left(-4\sum_{T-a}^{T}\lambda_{t}\right),$$

[10] $$\begin{array}{c} P\left(\mathrm{Monotypic}\right)=4\;\exp\left(-3\sum_{T-a}^{T}\lambda_{t}\right)\;\left(1-\exp\left(-\sum_{T-a}^{T}\lambda_{t}\right)\right). \end{array}$$

Here, $\lambda_{t}$ is the DENV serotype–specific transmission intensity in year t, assuming all four serotypes to circulate with equal intensities (34). T is the year at the time of giving birth and T-a the year the mother was born, with a representing the age of the mother. The probability of having experienced ≥2 DENV infections by year T is simply calculated as $1-P\left(\mathrm{Susceptible}\right)-P\left(\mathrm{Monotypic}\right)$. For each mother, we calculate a weighted mean expected DENV titer at the time of giving birth. We assume that DENV antibody titers are drawn from normal distributions, truncated between 0 and infinity. We assume that DENV PRNT titers of susceptible mothers have a mean of 20 and SD of 20, that monotypic titers have a mean of 150 and SD of 100, and that multitypic titers have a mean of 500 and SD of 200. As quantifying the specific titers linked to each serostatus is largely unknown and will differ by assay and cohort, we focus on the trends in titers in mothers and infants over time. We assign each birthing mother a mean DENV titer by drawing a random value from each of the three distributions and calculating the weighted mean titer, with weights equal to the respective probabilities of being susceptible, monotypic, and multitypic to DENV. We assume this titer to be equal to the maternally derived DENV antibody titer present in their infants at birth and subsequently model the decay of these titers in each infant assuming a constant exponential rate of decay as before (Eq. **2**). We use estimates of PRNT antibody half-life from the best-performing model as described above. Considering the same titer-related mechanisms of hospitalized infant dengue disease described above, the joint probability of an infant experiencing a DENV infection and risk of hospitalized disease given titer levels was calculated by age for each infant.

All codes used in the analyses are publicly available at https://github.com/meganodris/InfantDENV (35).

## Ethics Statement

The cohort study in Kamphaeng Phet was approved by Thailand Ministry of Public Health Ethical Research Committee, Siriraj Ethics Committee on Research Involving Human Subjects, Institutional Review Board for the Protection of Human Subjects State University of New York Upstate Medical University, and Walter Reed Army Institute of Research Institutional Review Board (protocol #2119). The Bangkok cohort study protocol was approved by the Ethics Committee of the Ministry of Public Health of Thailand. Our analysis is based on deidentified antibody titer data as well as deidentified hospital case data only.

Material has been reviewed by the Walter Reed Army Institute of Research. There is no objection to its presentation and/or publication. The opinions or assertions contained herein are the private views of the authors and are not to be construed as official, or as reflecting true views of the Department of the Army or the Department of Defense. The investigators have adhered to the policies for protection of human subjects as prescribed in AR 70–25.

## Supplementary Material

Appendix 01 (PDF)

## Data Availability

Anonymized (simulated data) data have been deposited in https://github.com/meganodris/InfantDENV (35).
