# Supplementary material for: Maternally derived antibody titer dynamics and risk of hospitalized infant dengue disease
Source: Proc Natl Acad Sci U S A. 2023 Sep 29;120(41):e2308221120. doi: 10.1073/pnas.2308221120 (PMC10576102; doi:10.1073/pnas.2308221120)
Supplement: Supplementary file 1 — Appendix 01 (PDF) [file pnas.2308221120.sapp.pdf]

Supplementary Information Appendix

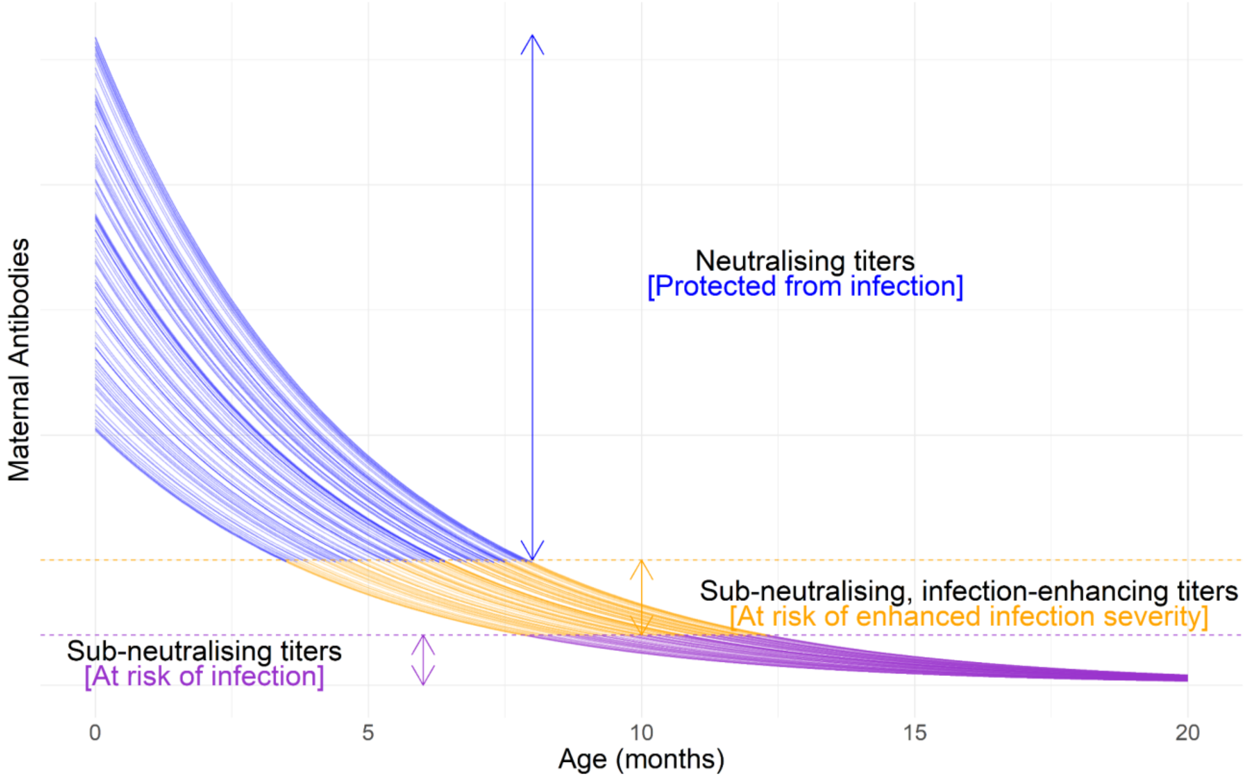

Figure S1. Illustration of maternally-derived DENV antibody decay dynamics and associated risks of infection and disease. Solid lines show the simulated maternal DENV antibody titers of 100 infants assuming an antibody half-life of 40 days. The colour of the lines indicate the expected risk profile of infants. Neutralising maternal antibody titers that protect infants against infection are shown in blue. Maternal antibody titers subsequently decay to levels that are sub-neutralising but infection-enhancing, shown in yellow, which place infants at risk of infection and at increased risk of severe disease, given infection. As maternal antibodies decay further, shown in purple, their ability to enhance heterologous infections is lost, reducing the risk of severe disease given infection, though infants will remain at risk of infection with these titer levels.

Model	Age (months)	Serotype	Half-life in days (95%CrI)			WAIC	ELPD LOO
			KPP HI	Bangkok HI	Bangkok PRNT		
Naïve	0-13	DENV1-4	79.1 (76.9-81.4)	79.1 (76.9-81.4)	79.1 (76.9-81.4)	16896.6	-8488.4
Base	0-13	DENV1-4	32.6 (31.5-33.6)	32.6 (31.5-33.6)	32.6 (31.5-33.6)	8167.7	-4183.2
Base-serotype	0-13	DENV1	32.7 (31.1-34.4)	32.7 (31.1-34.4)	32.7 (31.1-34.4)	8162.8	-4180.4
	0-13	DENV2	31.4 (29.7-33.1)	31.4 (29.7-33.1)	31.4 (29.7-33.1)		
	0-13	DENV3	32.2 (30.5-34.0)	32.2 (30.5-34.0)	32.2 (30.5-34.0)		
	0-13	DENV4	35.3 (32.9-37.8)	35.3 (32.9-37.8)	35.3 (32.9-37.8)		
Base-biphasic	0-3	DENV1-4	32.7 (31.6-33.7)	32.7 (31.6-33.7)	32.7 (31.6-33.7)	8164.6	-4179.2
	3-13	DENV1-4	33.0 (32.0-34.1)	33.0 (32.0-34.1)	33.0 (32.0-34.1)		
Naïve Cohort	0-13	DENV1-4	67.4 (63.4-72.0)	126.1 (120.8-131.9)	59.2 (57.0-61.5)	15501.1	-7793.7
Cohort	0-13	DENV1-4	38.5 (35.6-41.4)	47.0 (44.7-49.5)	28.1 (27.1-29.1)	7664.4	-3945.3
Cohort-serotype	0-13	DENV1	33.2 (28.5-38.8)	51.6 (46.8-56.9)	29.5 (27.9-31.3)	7667.5	-3951.8
		DENV2	41.2 (35.1-48.3)	45.1 (41.4-49.2)	26.9 (25.2-28.7)		
		DENV3	37.6 (32.9-43.0)	44.6 (40.9-48.7)	28.1 (26.3-30.1)		
		DENV4	40.9 (36.1-46.6)	48.2 (44.0-53.0)	27.0 (24.4-30.1)		
Cohort-biphasic	0-3	DENV1-4	35.5 (29.4-39.9)	46.6 (44.0-49.0)	27.5 (25.8-28.8)	7665.0	-3941.6
	3-13	DENV1-4	40.9 (36.7-48.9)	47.5 (45.1-50.1)	28.7 (27.5-30.4)		

Table S1. Maternal antibody decay model comparison. Median and 95% credible interval estimates of maternal antibody half-lives are shown for each of the considered models, along with their respective widely applicable information criterion (WAIC) and leave-one-out expected log pointwise predictive density (ELPD LOO) values.

Model	Data	Age (months)	Serotype	Half-life in days (95%CrI)		
				KPP HI	Bangkok HI	Bangkok PRNT
Cohort	Mean	0-13	DENV1-4	38.5 (35.6-41.4)	47.0 (44.7-49.5)	28.1 (27.1-29.1)
	Sero	0-13	DENV1-4	38.1 (35.7-40.4)	45.1 (42.5-47.7)	26.9 (25.8-28.0)
Cohort-Serotype	Mean	0-13	DENV1	33.2 (28.5-38.8)	51.6 (46.8-56.9)	29.5 (27.9-31.3)
			DENV2	41.2 (35.1-48.3)	45.1 (41.4-49.2)	26.9 (25.2-28.7)
			DENV3	37.6 (32.9-43.0)	44.6 (40.9-48.7)	28.1 (26.3-30.1)
			DENV4	40.9 (36.1-46.6)	48.2 (44.0-53.0)	27.0 (24.4-30.1)
	Sero	0-13	DENV1	34.7 (30.4-39.4)	49.1 (43.8-55.0)	28.2 (26.4-30.1)
			DENV2	38.3 (33.3-43.9)	43.3 (39.2-47.8)	26.6 (24.7-28.8)
			DENV3	34.8 (31.1-38.9)	43.2 (39.2-47.7)	26.4 (24.3-28.6)
			DENV4	42.3 (38.4-46.6)	45.6 (41.0-50.8)	24.3 (21.5-27.6)

Table S2. Maternal antibody decay model comparison with different infection exclusion criteria. Median and 95% credible interval estimates of maternal antibody half-lives are shown for each of the considered models. Models were fit using data where samples with mean increases in titers across serotypes were excluded as potential infections [mean] and where samples with serotype-specific increases in titers were excluded as potential infections [sero].

Model	Cohort [Assay]	Median parameter estimates (95%CrI)			Fixed δ	WAIC	ELPD LOO
		μ	σ	δ			
Normal (δ=0)	KPP [HI]	6.1 (5.5-6.6)	2.8 (2.5-3.2)	-	0	486.8	-244.5
	Bangkok [HI]	3.4 (3.1-3.6)	1.4 (1.3-1.6)	-			
	Bangkok [PRNT]	8.2 (7.1-9.7)	4.3 (3.7-5.2)	-			
Normal + Age effect (δ=0.05)	KPP [HI]	5.4 (4.8-5.9)	2.7 (2.4-3.1)	-	0.05	500.7	-250.5
	Bangkok [HI]	3.0 (2.8-3.2)	1.4 (1.2-1.5)	-			
	Bangkok [PRNT]	6.8 (6.0-7.8)	3.9 (3.4-4.6)	-			
Normal + Age effect (δ=0.15)	KPP [HI]	4.0 (3.6-4.4)	2.5 (2.2-3.1)	-	0.15	531.6	-268.6
	Bangkok [HI]	2.2 (2.0-2.4)	1.4 (1.3-1.6)	-			
	Bangkok [PRNT]	4.7 (4.2-5.2)	3.4 (3.1-4.1)	-			
Normal + Age effect (δ=0.25)	KPP [HI]	2.7 (2.3-3.0)	2.5 (2.2-2.9)	-	0.25	577.8	-286.7
	Bangkok [HI]	0.6 (0.1-1.2)	1.9 (1.5-2.2)	-			
	Bangkok [PRNT]	2.2 (1.6-2.7)	2.8 (2.3-3.4)	-			
Normal + Age effect	KPP [HI]	5.8 (5.1-6.5)	2.8 (2.4-3.2)	0.02 (0.00-0.05)	-	499.0	-248.1
	Bangkok [HI]	3.2 (2.9-3.6)	1.4 (1.3-1.6)				
	Bangkok [PRNT]	7.7 (6.5-9.2)	4.2 (3.5-5.1)				
Lognormal (δ=0)	KPP [HI]	2.1 (1.9-2.2)	1.0 (0.9-1.1)	-	0	387.4	-194.4
	Bangkok [HI]	1.3 (1.2-1.4)	0.8 (0.7-0.8)	-			
	Bangkok [PRNT]	3.0 (2.7-3.3)	1.4 (1.3-1.5)	-			
Lognormal + Age effect (δ=0.05)	KPP [HI]	1.9 (1.7-2.0)	1.0 (0.9-1.0)	-	0.05	401.3	-201.4
	Bangkok [HI]	1.1 (1.0-1.2)	0.8 (0.7-0.8)	-			
	Bangkok [PRNT]	2.6 (2.3-2.9)	1.4 (1.3-1.5)	-			
Lognormal + Age effect (δ=0.15)	KPP [HI]	1.4 (1.3-1.5)	0.9 (0.8-1.0)	-	0.15	433.2	-217.2
	Bangkok [HI]	0.7 (0.6-0.8)	0.8 (0.7-0.8)	-			
	Bangkok [PRNT]	1.8 (1.6-2.1)	1.3 (1.2-1.4)	-			

Lognormal + Age effect (δ=0.25)	KPP [HI]	1.0 (0.9-1.1)	0.9 (0.8-1.0)	-	0.25	467.3	-235.2
	Bangkok [HI]	0.3 (0.3-0.4)	0.8 (0.7-0.9)	-			
	Bangkok [PRNT]	1.1 (0.9-1.3)	1.3 (1.2-1.4)	-			
Lognormal + Age effect	KPP [HI]	2.1 (1.9-2.2)	1.0 (0.9-1.1)	0.01 (0.00-0.03)	-	389.7	-195.5
	Bangkok [HI]	1.3 (1.1-1.4)	0.8 (0.7-0.8)				
	Bangkok [PRNT]	2.9 (2.6-3.2)	1.4 (1.3-1.5)				
Threshold (δ=0)	KPP [HI]	24.3 (21.8-27.6)	0.6 (0.0-2.0)	-	0	820.3	-414.9
	Bangkok [HI]	9.1 (8.7-9.3)	0.1 (0.0-0.3)	-			
	Bangkok [PRNT]	50.4 (44.3-57.5)	0.6 (0.0-2.0)	-			
Threshold + Age effect (δ=0.05)	KPP [HI]	21.3 (17.9-24.1)	0.7 (0.0-2.2)	-	0.05	668.4	-335.0
	Bangkok [HI]	8.9 (8.4-9.3)	0.1 (0.0-0.4)	-			
	Bangkok [PRNT]	42.1 (34.9-47.6)	0.7 (0.0-2.2)	-			
Threshold + Age effect (δ=0.15)	KPP [HI]	11.6 (10.2-13.5)	1.3 (0.1-2.7)	-	0.15	548.1	-276.0
	Bangkok [HI]	5.7 (5.3-5.9)	0.2 (0.0-0.8)	-			
	Bangkok [PRNT]	11.8 (11.2-11.9)	0.0 (0.0-0.3)	-			
Threshold + Age effect (δ=0.25)	KPP [HI]	8.1 (7.3-8.7)	0.14 (0.0-0.7)	-	0.25	538.2	-272.3
	Bangkok [HI]	3.8 (3.5-4.1)	1.3 (1.0-1.5)	-			
	Bangkok [PRNT]	11.1 (10.2-11.7)	0.1 (0.0-0.6)	-			
Threshold + Age effect	KPP [HI]	8.6 (8.1-8.8)	0.1 (0.0-0.4)	0.21 (0.19-0.22)	-	509.5	-260.5
	Bangkok [HI]	4.1 (3.8-4.5)	1.3 (1.0-1.6)				
	Bangkok [PRNT]	11.4 (10.8-11.8)	0.1 (0.0-0.4)				

Table S3. Titer-related mechanisms of hospitalised disease model comparison. Median and 95% credible interval estimates of parameter are shown for each of the considered models, along with their respective widely applicable information criterion (WAIC) and leave-one-out expected log pointwise predictive density (ELPD LOO) values.

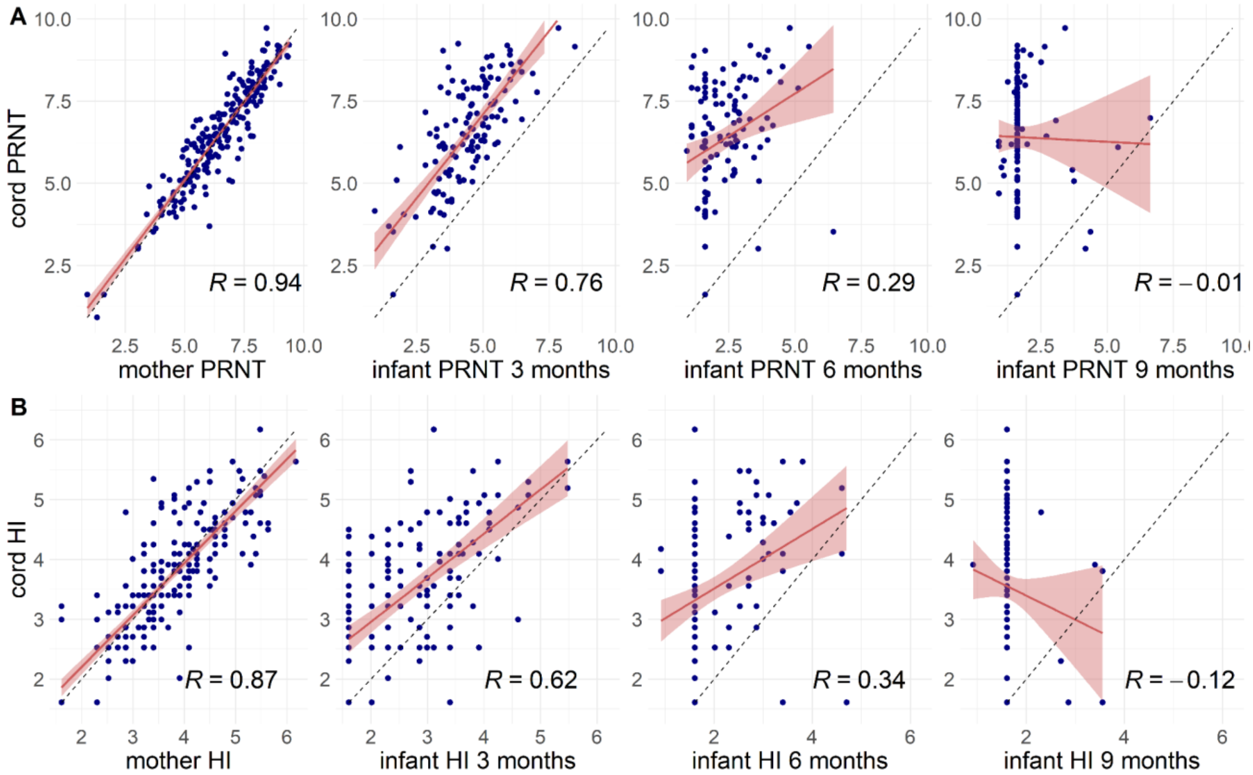

Figure S2. Correlation in cord titers at birth with mother's titers and infant titers by age in the Bangkok cohort for both PRNT (A) and HI (B) assays. Blue points show individual PRNT and HI titer values on a log scale. The red line and ribbon show the mean and 95% confidence intervals of a linear regression model fitted to these data and corresponding Pearson correlation coefficients, R, are shown in black. The black dashed line indicates where log cord titers would be equal to mother or infant titers.

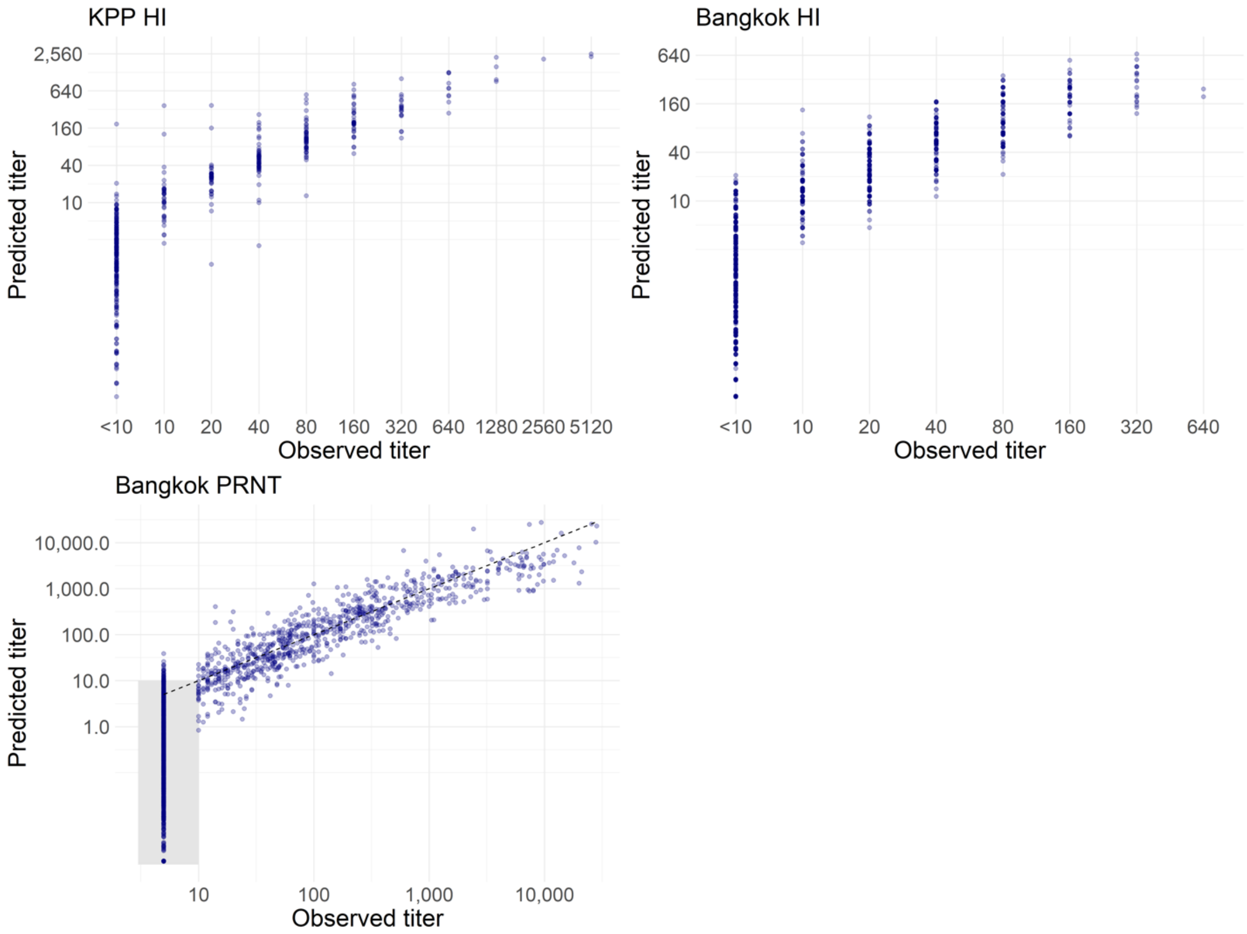

Figure S3. Observed vs Model Predicted titer. Blue points show the observed vs model predicted antibody titer estimates for the best performing cohort model. The dashed line indicates the ideal scenario of predicted=observed PRNT titers and the grey shaded area indicates the titer values below the lower limit of detection of the PRNT assay.

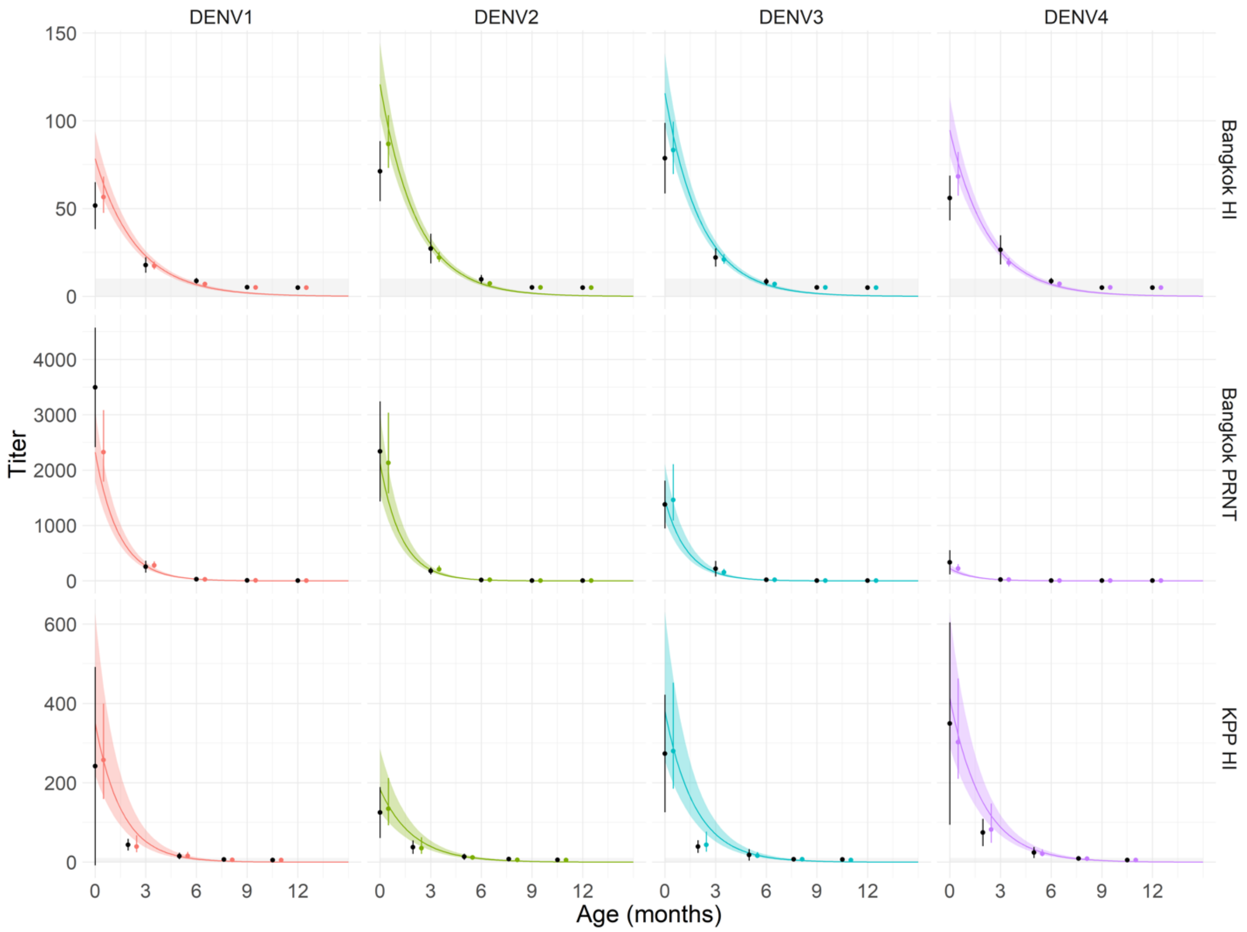

Figure S4. Model fit for the cohort-serotype model. Coloured lines and ribbons show the median and 95% credible interval estimates of population-level serotype-specific maternal DENV antibody titers. Black points and lines indicate the population mean and 95% confidence interval titers observed in the data. Coloured points and lines show the median and 95% credible interval model estimated population mean titers, transformed to the respective discretized measurement processes of each assay. Grey shaded areas indicate the lower limits of assay detection.

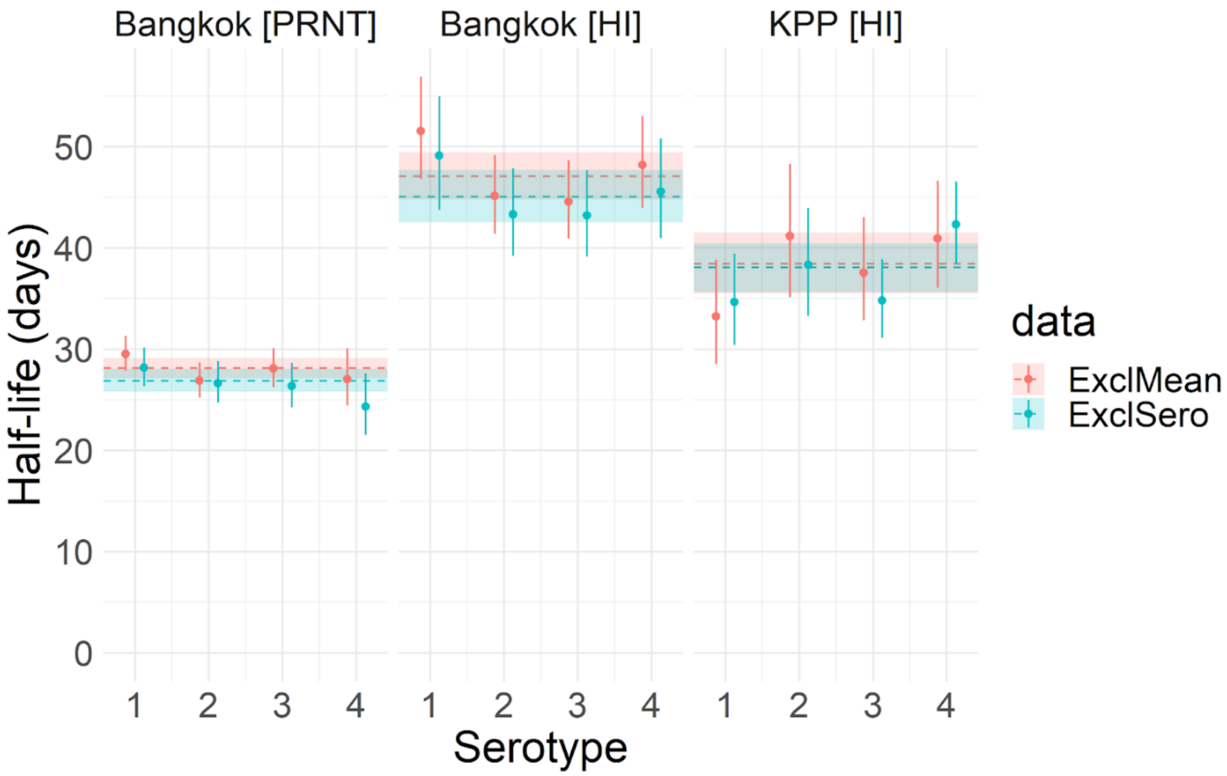

Figure S5. Estimates of mean and serotype-specific antibody half-lives using different data exclusion criteria. Coloured points and lines indicate median and 95% credible interval antibody half-life estimates by serotype (from the cohort-serotype model). The dashed lines and shaded ribbons show the median and 95% credible interval estimates of mean antibody half-life across serotypes (from the cohort model). Colours indicate whether infant samples with increases in their mean titers across serotypes (ExclMean) or with increases in serotype-specific titers (ExclSero) were excluded from the analysis.

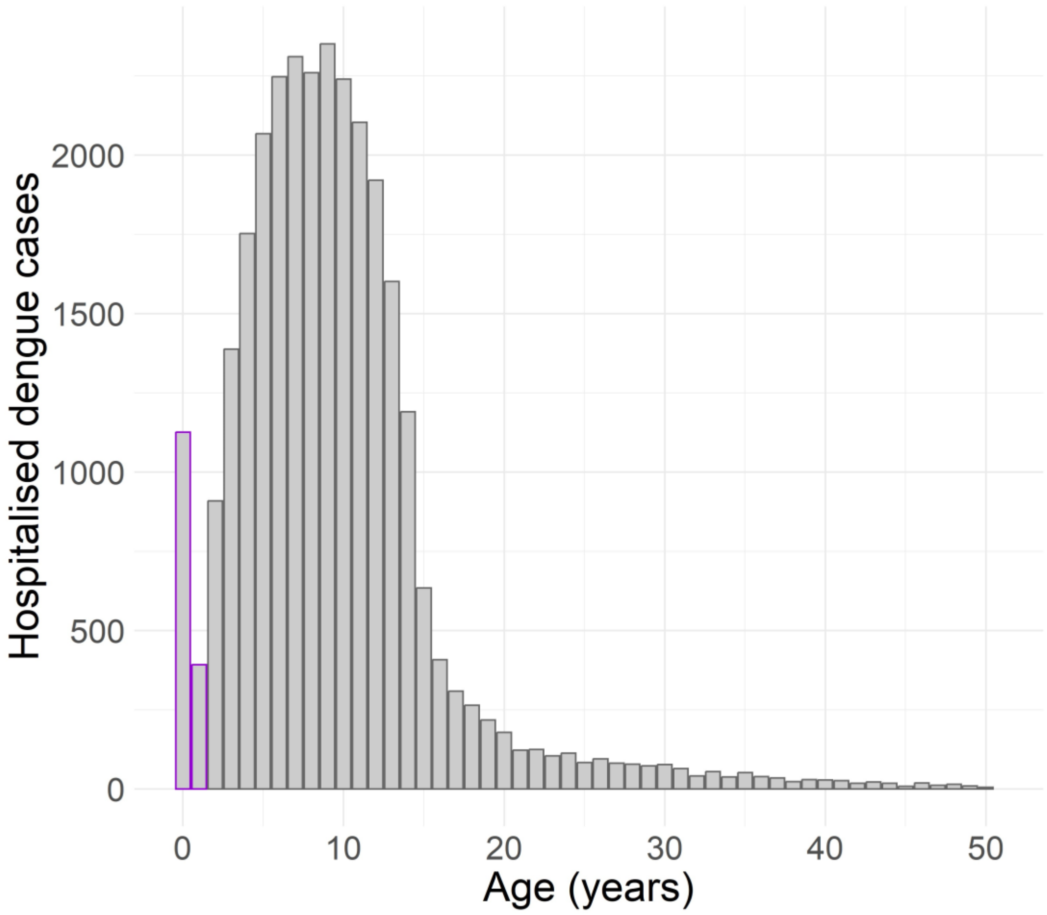

Figure S6. Age distribution of hospitalised dengue cases in Queen Sirikit National Institute of Child Health and Kamphaeng Phet hospitals. Grey bars show the cumulative number of hospitalised dengue cases reported during this time period (1974-2014 for QSNICH and 1994-2019 for Kamphaeng Phet hospital). Bars highlighted in purple indicate hospitalised dengue cases aged 0-1 and 1-2 respectively.

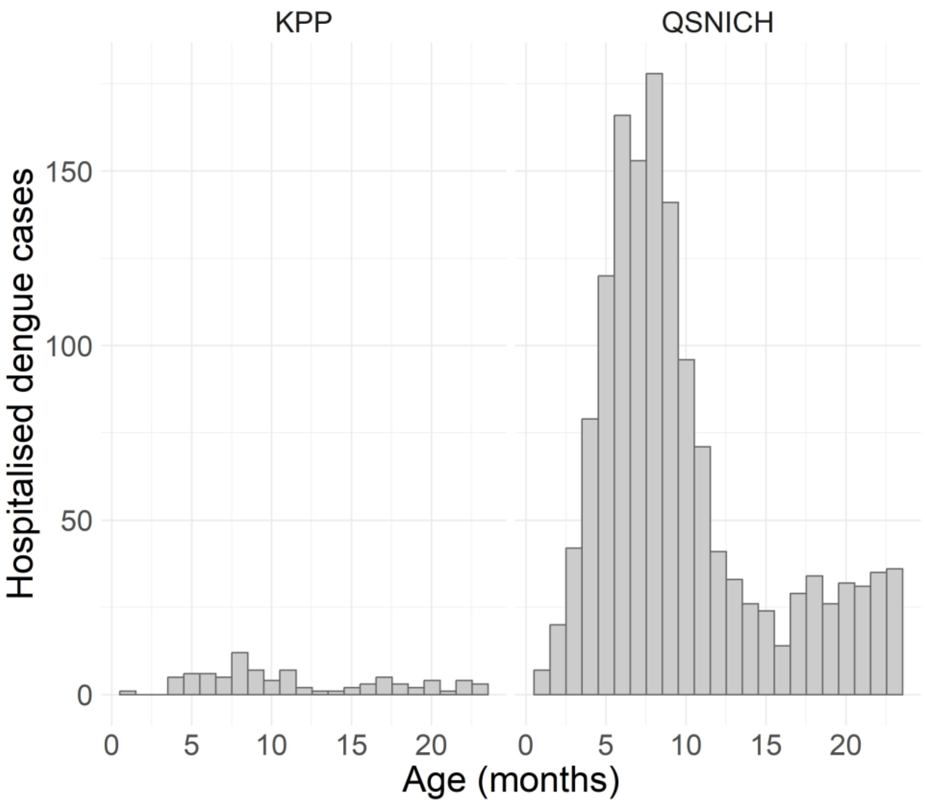

Figure S7. Age distribution of hospitalised infant dengue cases by reporting hospital. Data from Kamphaeng Phet (KPP) hospital was available for the period 1994-2019 and for the period 1974-2014 from Queen Sirikit National Institute of Child Health (QSNICH).

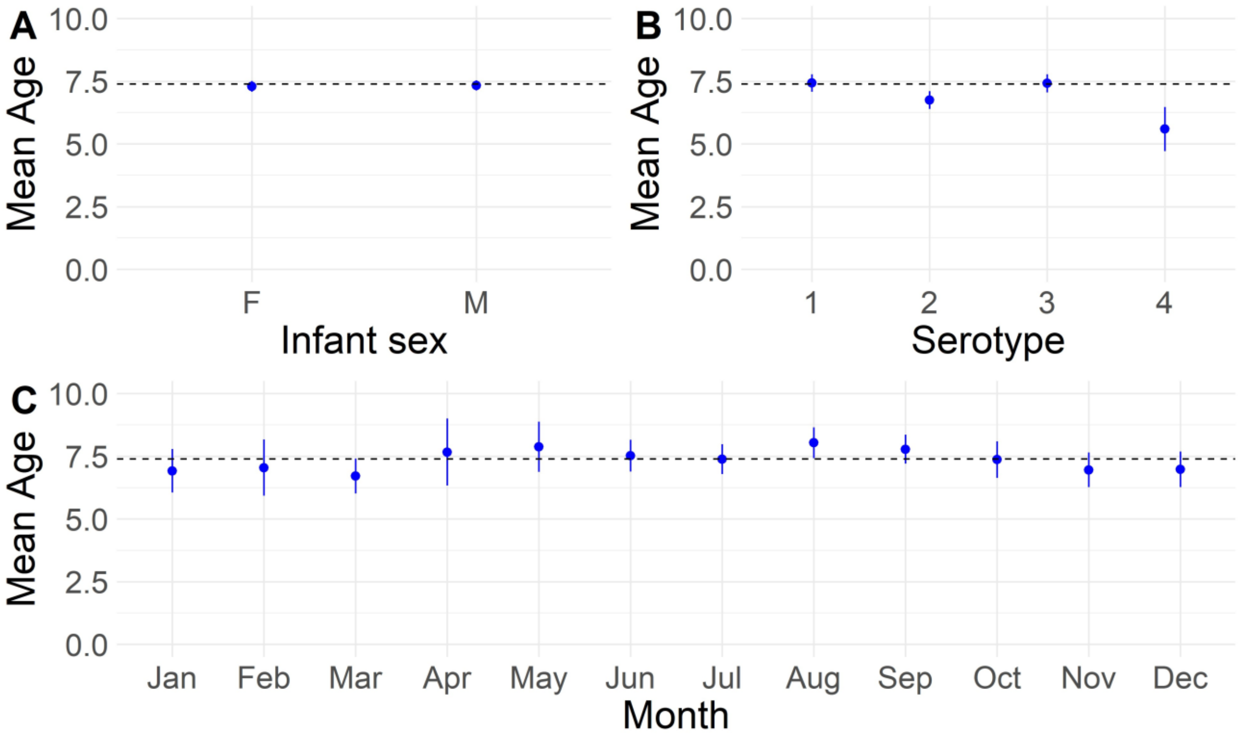

Figure S8. Mean age, in months, of hospitalised infant dengue cases by sex (A), infecting

serotype (B) and by month of hospitalisation (C). Blue points and lines indicate the mean and 95% confidence interval. Black dashed lines indicate the overall mean age of infant hospitalised dengue cases.

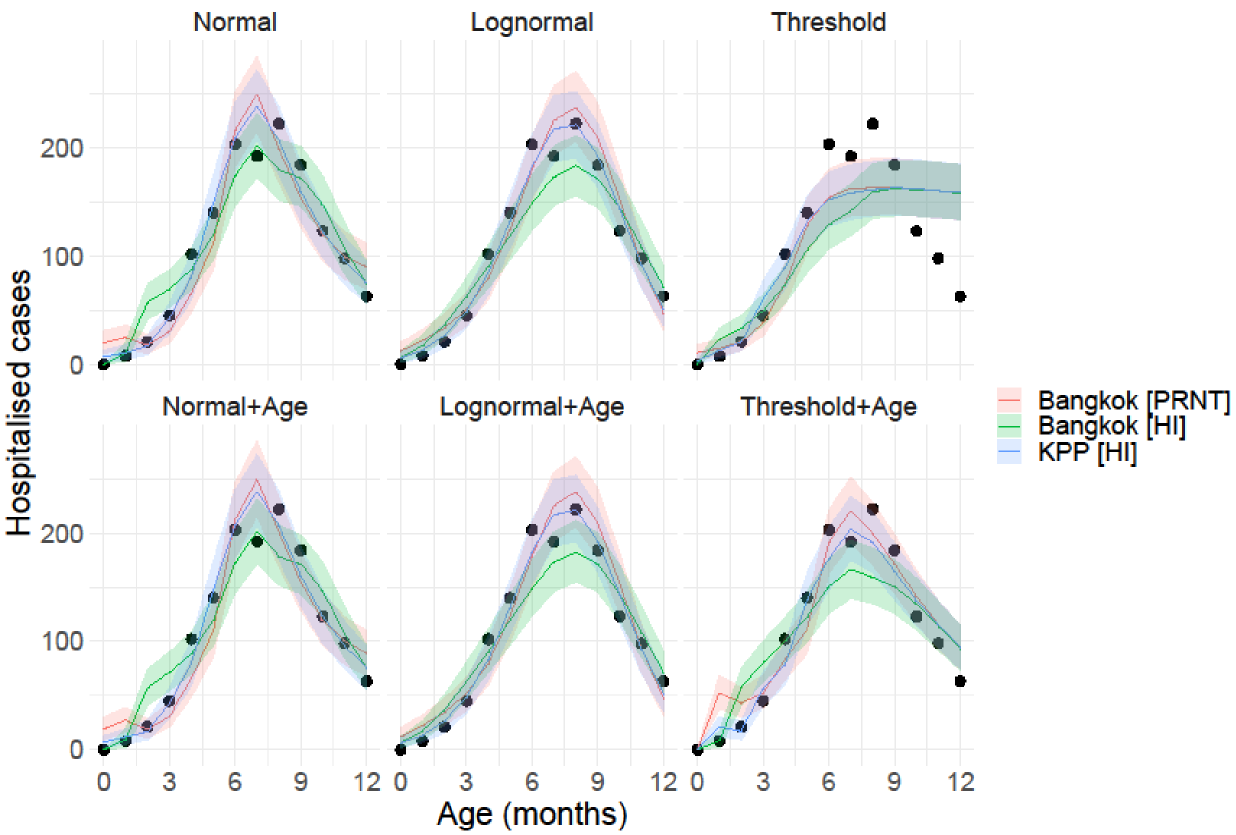

Figure S9. Model fits to the age distribution of hospitalised infant dengue cases. Black points show the reported number of cases by age. Coloured lines and shaded ribbons show the median and 95% credible interval estimates of age-specific hospitalised dengue cases. Each panel shows the results from different model parameterizations and colours indicate which antibody titer data was used in the model.

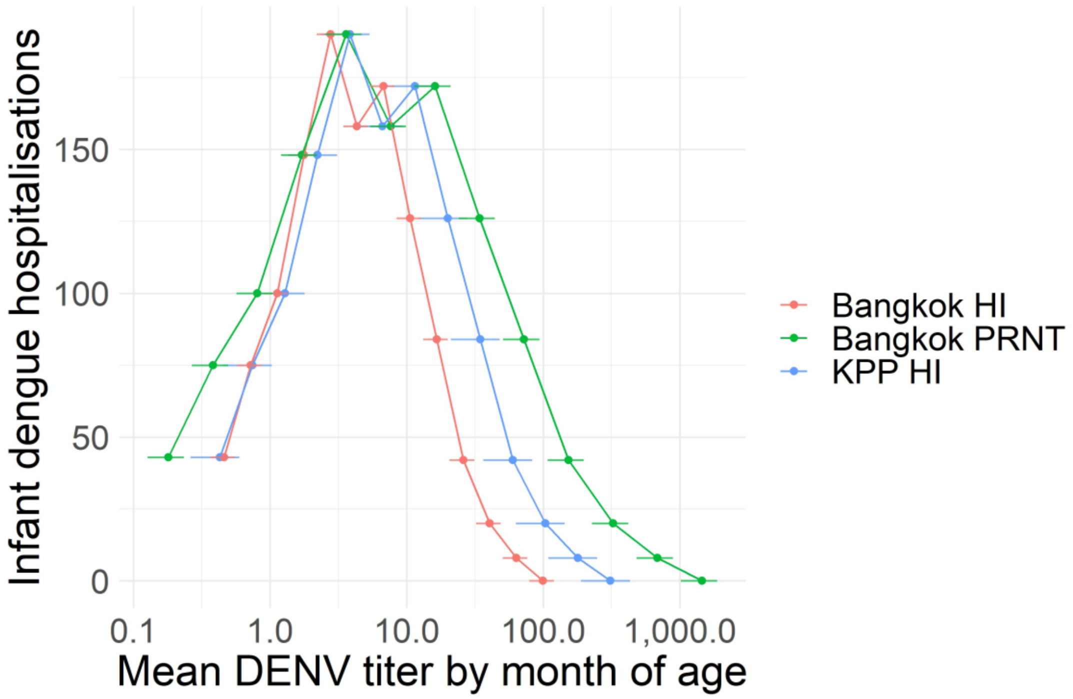

Figure S10. Estimated mean maternally-derived DENV titer by month of infant age and reported number of infant dengue hospitalisations by the corresponding month of infant age. The coloured points and horizontal lines indicate the estimated mean and 95% confidence interval infant maternally-derived DENV titer across infants at each month of infant age, up to 13 months. Colours indicate which cohort and assay the titer estimates correspond to.

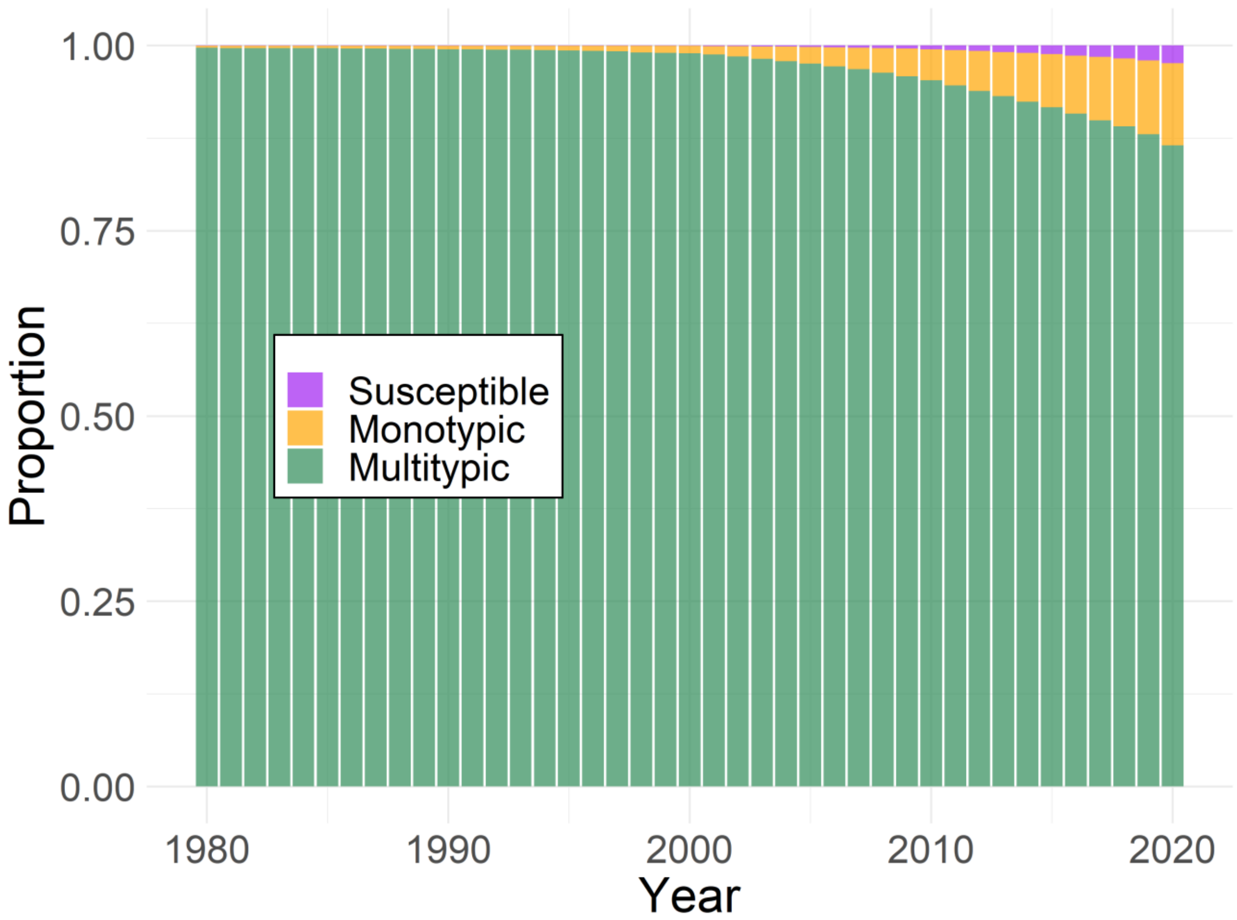

Figure S11. Simulated proportion of birthing mothers in Thailand that are susceptible, monotypic immune and multitypic immune to dengue virus by year, 1980-2019.

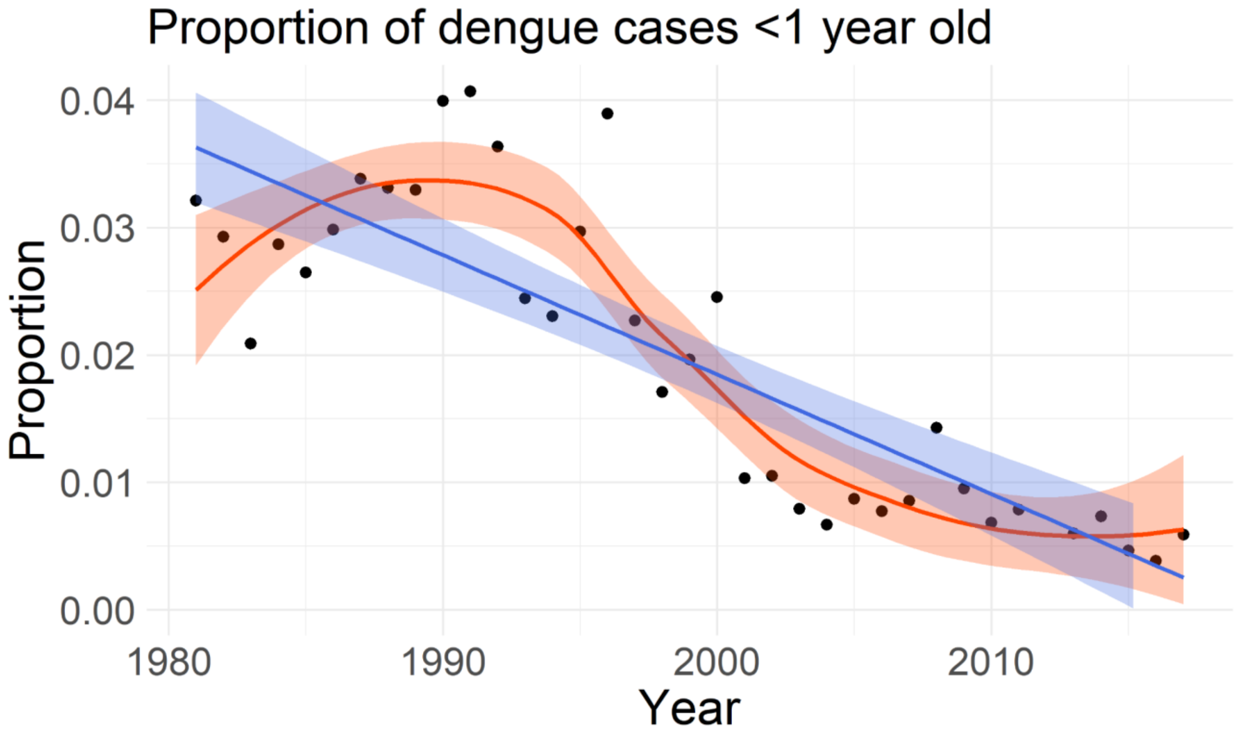

Figure S12. Annual proportions of all dengue hospitalisations reported in Bangkok that were attributed to infants <1 year of age between 1981 and 2017. Black points show the annual proportions of infant hospitalisations. The blue line and shaded ribbon show the mean and 95% confidence interval fit of a linear regression model, while the orange line and ribbon show the mean and 95% confidence interval fit of a loess model to the observed data.

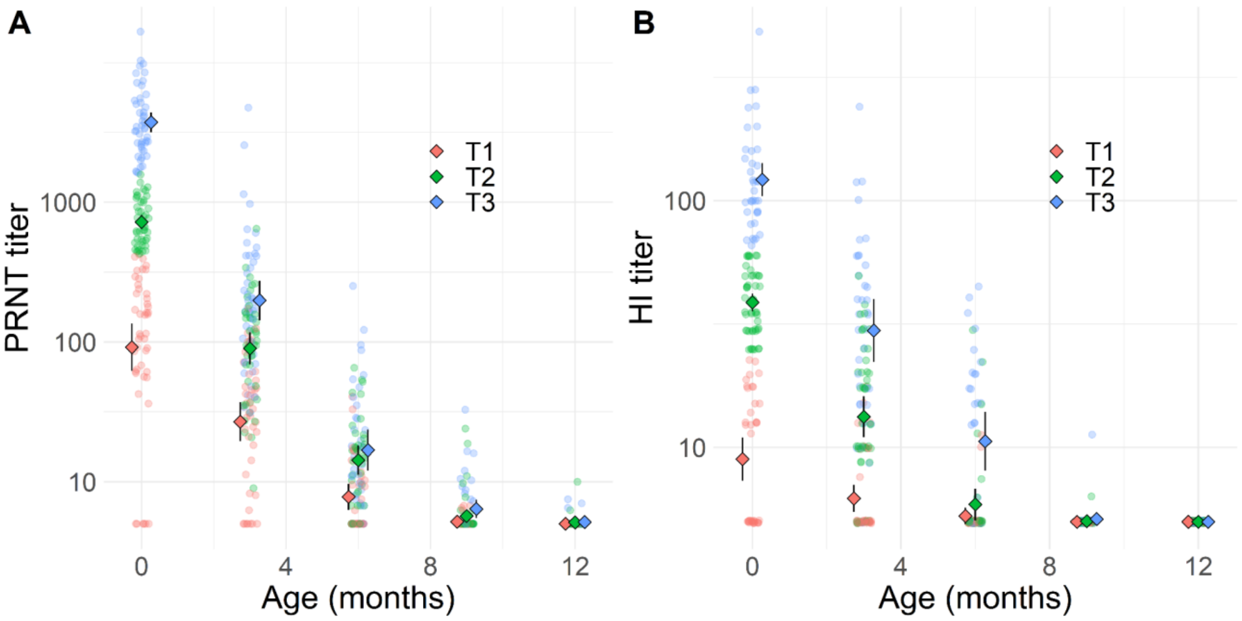

Figure S13. Geometric mean titers by age grouped by tertiles of cord blood titers at

birth in the Bangkok cohort for PRNT (A) and HI (B) assays. Shaded points show individual DENV titers by age, averaged across the 4 serotypes, with colours indicating the cord blood tertile group of each individual. Coloured diamonds and black lines indicate the group-specific geometric mean titers by age and corresponding 95% confidence intervals.

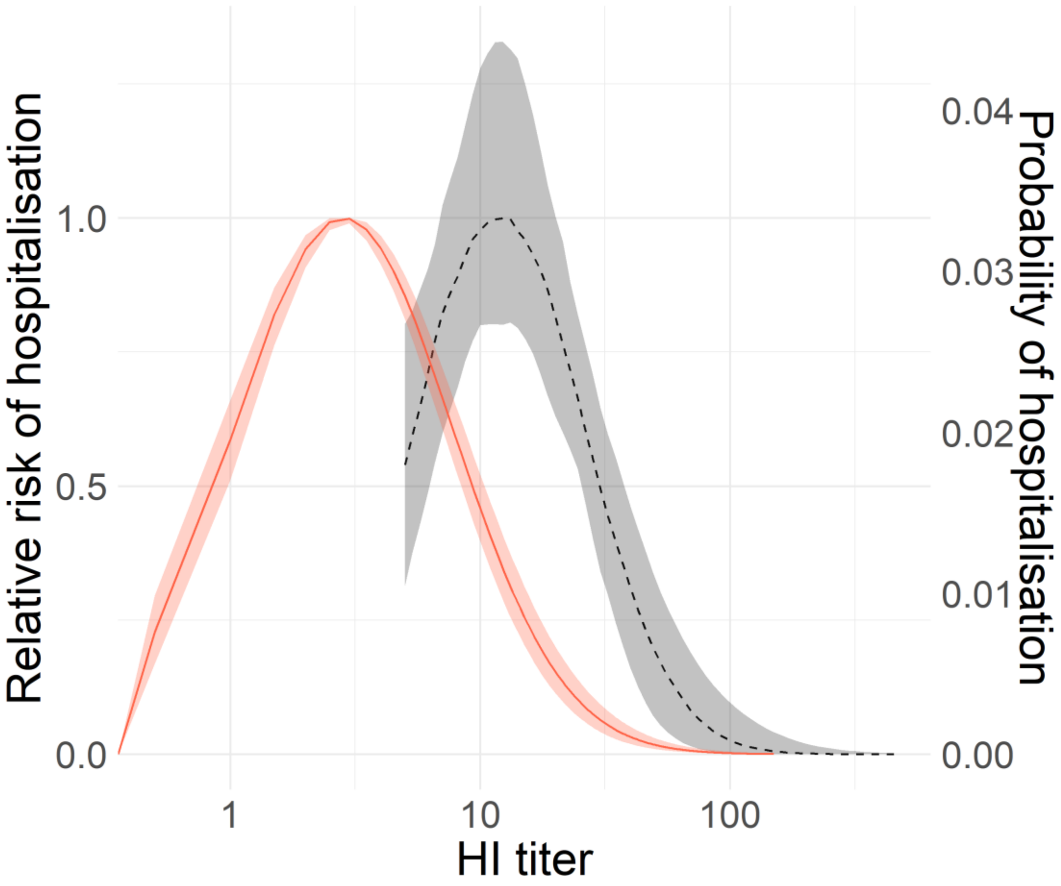

Figure S14. Estimated titer distributions associated with the age of increased risk of hospitalised dengue disease in Kamphaeng Phet cohorts. The pink line and shaded ribbon shows median and 95% credible interval model estimates of the relative risk of hospitalised infant disease given a DENV infection by HI titer considering the lognormal ADE scenario. The grey dashed line and shaded ribbon show the median and 95% credible interval estimates of the probability of dengue hospitalisation by HI titer in the wider Kamphaeng Phet cohort (children and adults), estimated in Salje et al., corresponding to the y-axis on the right hand side of the panel (1).

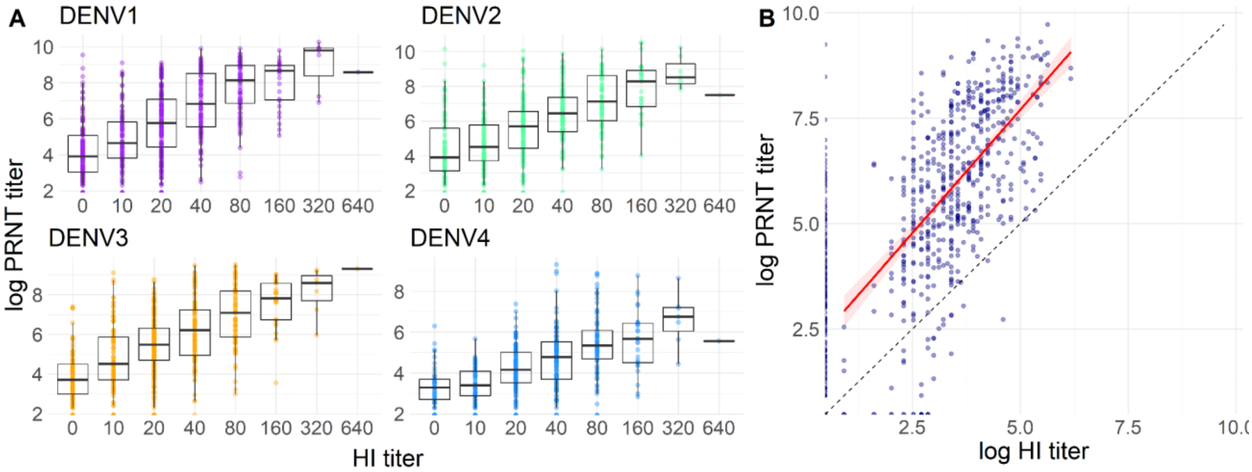

Figure S15. Correlations in PRNT and HI titers in the Bangkok cohort. (A) Coloured points show individual-level HI and log PRNT titers by serotype. Boxplots show the median and interquartile range of log PRNT titers per HI reading. (B) Blue points show individual-level log HI and PRNT titers averaged across serotypes, with the red line and ribbon showing the mean and 95% confidence interval linear regression model fitted to these data points (Pearson correlation coefficient=0.62 (95%CI: 0.58-0.65)). The black dashed line indicates where log HI values would be equal to log PRNT values.

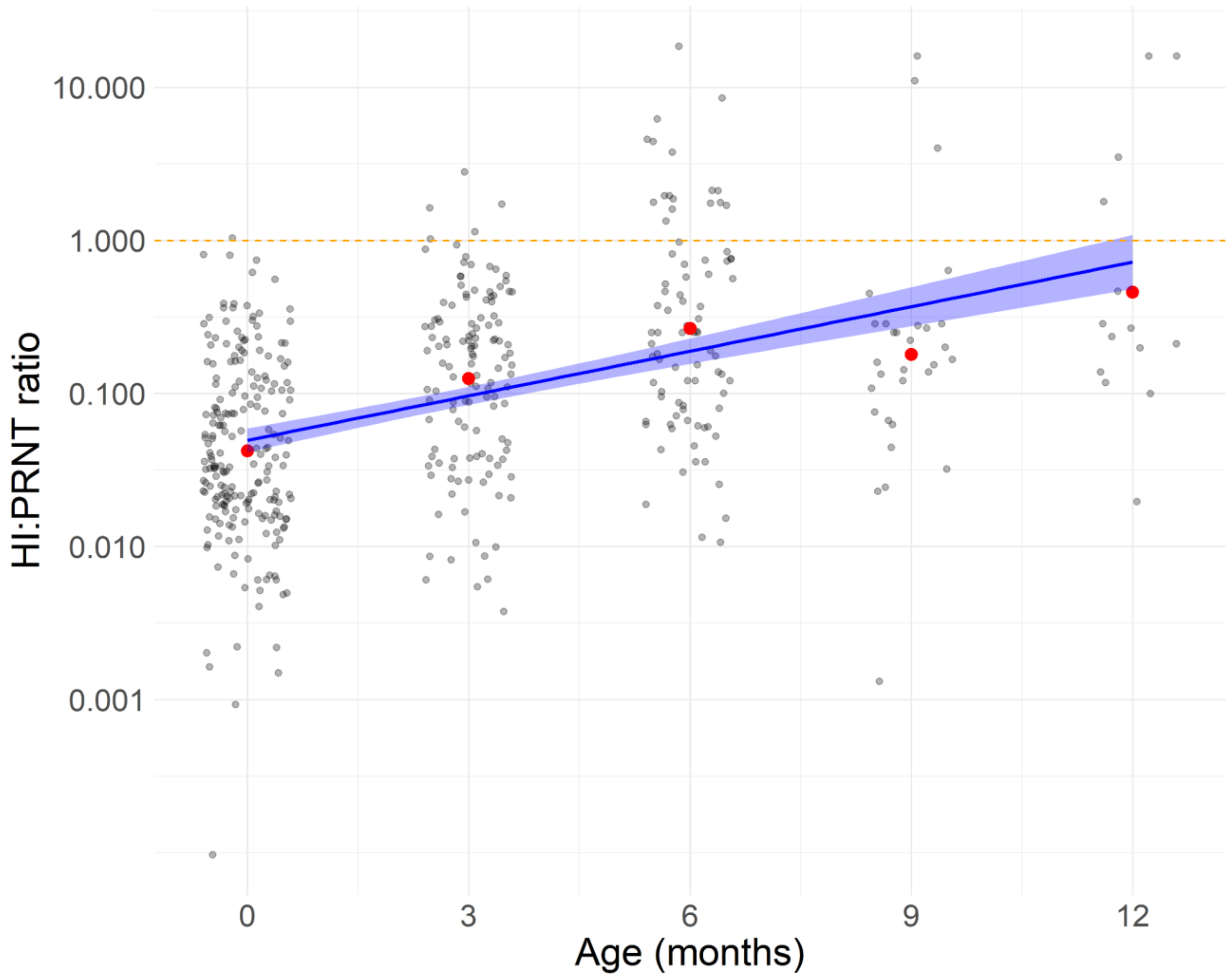

Figure S16. HI:PRNT ratios observed in the Bangkok cohort by month of age. Black points show individual infant HI:PRNT ratios and red points show the mean ratio by each age group. The blue line and ribbon show the mean and 95% confidence interval fit of a linear model to the observed ratios. The orange dashed line at 1 indicates where HI and PRNT titers become equivalent.

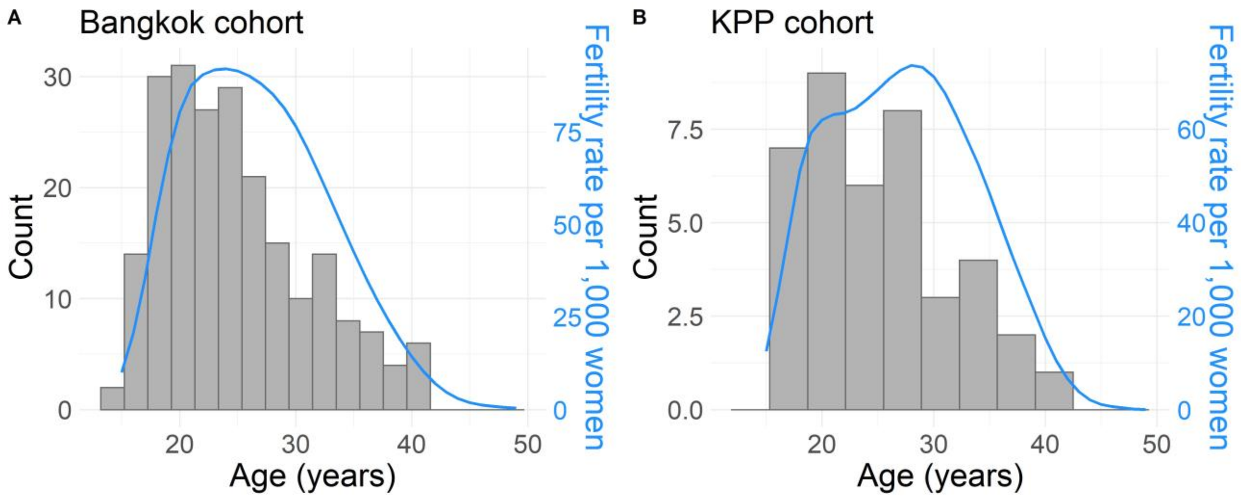

Figure S17. Age distribution of birthing women in each cohort compared to fertility rates of the Thai population. Grey bars show the number of birthing women by age in the Bangkok cohort (panel A) and Kamphaeng Phet cohort (panel B). Blue lines show the mean age-specific fertility rates per 1,000 women in Thailand over the same time periods as the cohort studies (2000-2003 for Bangkok and 2015-2021 for Kamphaeng Phet cohorts) (2).

References:

1. H. Salje, *et al.*, Reconstruction of antibody dynamics and infection histories to evaluate dengue risk. *Nature* **557**, 719–723 (2018).

2. United Nations, World Population Prospects - Population Division - United Nations (2022) (June 30, 2022).
